# Path analysis model to identify the effect of poor diet quality on NAFLD among Iranian adults from Amol Cohort Study

**DOI:** 10.1038/s41598-024-70181-4

**Published:** 2024-08-27

**Authors:** Azam Doustmohammadian, Bahareh Amirkalali, Barbora de Courten, Saeed Esfandyari, Nima Motamed, Mansooreh Maadi, Hossein Ajdarkosh, Esmaeel Gholizadeh, Samira Chaibakhsh, Farhad Zamani

**Affiliations:** 1 Gastrointestinal and Liver Diseases Research Center, Iran University of Medical Sciences, Tehran, Iran; 2 School of Health and Biomedical Sciences, RMIT University, Melbourne, VIC 3085 Australia; 3 Asadabad School of Medical Sciences, Hamadan, Iran; 4 Department of Social Medicine, Zanjan University of Medical Sciences, Zanjan, Iran; 5 Rajaie Cardiovascular Medical and Research Center, Iran University of Medical Sciences, Tehran, Iran

**Keywords:** Diet, Nonalcoholic Fatty Liver Disease, CRP, Hemoglobin A1c protein, Iran, Non-alcoholic fatty liver disease, Risk factors

## Abstract

Nonalcoholic fatty liver disease (NAFLD) is expanding as a global health problem with approximately 25% of the world's population affected by it. Dietary modification is one of the most important strategies for preventing NAFLD. The association between nutrient density and the Healthy Eating Index 2015 (HEI2015) with NAFLD demonstrates that nutrient density is an independent predictor of NAFLD in Iranian adults [fully adjusted model: OR (95% CI)_tertile3vs.1_: 0.68 (0.54–0.85), P _for trend_ = 0.001]. However, a favorable association between NAFDL and diet quality (HEI 2015) is more pronounced in participants with abdominal obesity [fully adjusted model: OR (95% CI)_tertile3vs.1_: 0.63 (0.41–0.98), P _for trend_ = 0.03]. Based on the gender-stratified path analysis, diet quality indirectly through Waist-to-Height Ratio (WHtR), C-reactive protein (CRP), and metabolic syndrome in women, and men through WHtR, hemoglobin A1c (HBA1c), CRP, and metabolic syndrome affects NAFLD. Nutrient density directly and indirectly in women through WHtR, CRP, and metabolic syndrome, and in men indirectly through WHtR, hemoglobin A1c, and metabolic syndrome negatively affect NAFLD. Hence, in these subjects; we can provide early dietary intervention and education to prevent progression to NAFLD.

## Introduction

Nonalcoholic fatty liver disease (NAFLD) is expanding as a global health problem in developed and developing countries^1^ with approximately 25% of the world's population affected by it. NAFLD and its progression can lead to liver cirrhosis or even liver cancer^2^. By 2025, it will be the major cause of liver transplants in America ^3^. Studies also provide evidence that NAFLD may operate as a stand-alone risk factor for cardiovascular disease mortality and morbidity^4^, therefore, it is important to understand the risk factors for NAFLD.

In the past, this disease was defined as the accumulation of fat in the liver, which was diagnosed by imaging methods or pathology, without excessive alcohol consumption or any other causes resulting in secondary hepatic fat infiltration^5,6^. But recently, a new definition has been considered, describing NAFLD as a metabolic-associated fatty liver disease (MAFLD) disease. In this new definition, besides the accumulation of fat in the liver, one of the following three factors is also necessary for diagnosing the disease: obesity or overweight, type 2 diabetes, or evidence of metabolic disorders^7^. This new definition shows a clearer picture of the relationship between this disease and many lifestyle-related factors.

Lifestyle modification is the first line of treatment for NAFLD, and adherence to a healthy diet is one of the main treatment pillars^8^. Studies have shown that improving diet quality plays an important role in the prevention and treatment of NAFLD. Diet can be effective through weight control, treatment of metabolic syndrome and cardiovascular risk factors, and improving the antioxidant capacity of the body^9^.

There are different indicators for examining diet quality^10,11^. Two of these indicators are Healthy Eating Index 2015 (HEI_2015_)^12^ and Nutrient-Rich Food 9.3. (NRF_9.3_) scores that can give us an accurate estimation of diet quality^13^.

HEI_2015_ index examines dietary adherence of people to the American Dietary Guideline (ADG)^14^, and previous studies indicate that the improvement of this index is associated with an improvement of NALD as well as the risk of cardiovascular diseases, cancer, type 2 diabetes, all-cause mortality^15,16^. NRF_9.3_ index, which is used to measure nutrient density in the diet, includes three nutrients to limit (saturated fat, added sugar, sodium) and nine nutrients to encourage (protein, fiber, vitamins A, C, D, calcium, magnesium, potassium, and iron). The results of the evidence show that affordable nutrient-rich dietary patterns are linked to preventing disease and/or positively influencing health.

NAFLD is a multifactorial disease. Besides diet, various risk factors, such as demographic, environmental, lifestyle, and clinical factors, contribute to its development and progression.^17,18^. NAFLD has garnered attention for its associations with metabolic abnormalities and cardiovascular risks. Specifically, C-reactive protein (CRP) and glycated hemoglobin (HbA1c) have been of interest as potential biomarkers for systemic inflammation and dysregulated glucose metabolism, respectively^19–21^. Extensive research has focused on the relationship between NAFLD and metabolic syndrome, a cluster of conditions like obesity, hypertension, dyslipidemia, and insulin resistance. Those with metabolic syndrome are more likely to develop NAFLD, emphasizing the complex interaction between liver fat buildup and metabolic irregularities^22^. Additionally, the impact of lifestyle choices, such as physical activity, on NAFLD risk has been a key area of investigation^23^. Studies suggest that regular exercise can mitigate hepatic fat accumulation and improve insulin sensitivity, potentially serving as a therapeutic strategy for managing NAFLD^24^.

**Figure 1:**
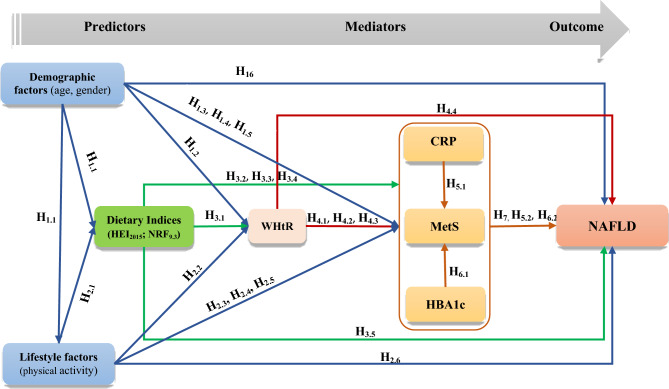
The research conceptual framework of the relationship between Dietary Indices (HEI_2015_ & NRF_9.3_) and NAFLD considering the effect of mediators and other variables.

Based on the literature review (Supplementary file 1), we proposed a schematic pathway involving interactions among HEI_2015_, NRF_9.3_, NAFLD, and possible mediating variables such as anthropometric parameters, and cardiometabolic risk factors (Fig. 1).

It is important to understand how these factors interact with each other to cause NAFLD to design the most effective interventions.

**Figure 2:**
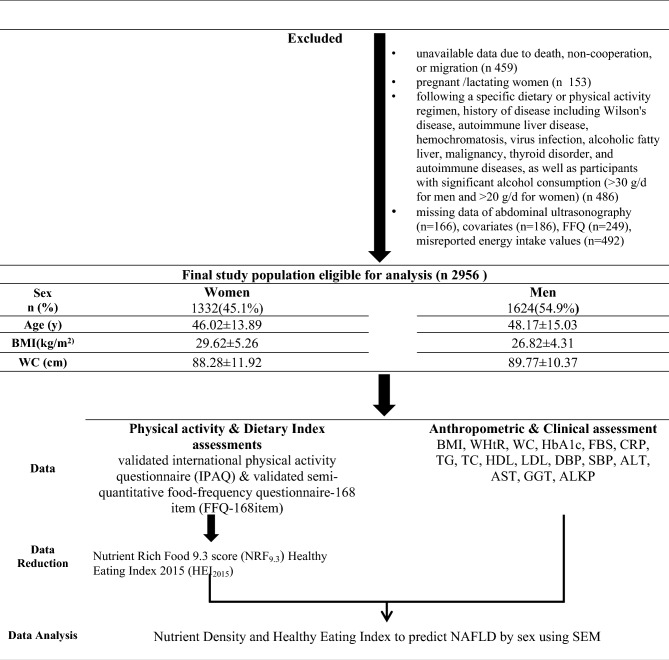
Flowchart of the study design.

Path analysis appears well-suited to address the limitations of multiple regression models. This method places all effective, independent and dependent variables in a model, simultaneously examines how these variables relate to the outcome, detects missing paths, and computes indices for overall goodness of fit^25^. Path analysis has been used in a few studies on the association between diet quality and NAFLD^26^. Due to the diversity in dietary patterns of different countries, in this study, we intended to use the Path analysis method to evaluate the association between HEI_2015_, NRF_9.3_, NAFLD, and possible mediating factors in a community-based cross-sectional study, Amol, Iran (Fig. 2).

## Results

### Baseline characteristics of the study participants

Of 2956 (47.09 ± 14.46) recruited in the cohort, 1332 (45.1%) were women. The prevalence of NAFLD was 46% (n = 1360). Details of socio-demographic, anthropometric, biochemical parameters, physical activity and dietary assessment according to gender and disease status are presented in Tables 1 and 2. NAFLD participants had a higher BMI, WC, and WHtR compared to healthy adults. Compared to those without NAFLD, diabetes and metabolic syndrome comorbidities were much more highly prevalent in patients with NAFLD (all P < 0.001). In women with NAFLD, the medication history approached significance. This was significant for men who used glucose-lowering agents (p = 0.001). In both genders, there were high levels of TG, total cholesterol, and LDL in patients with NAFLD, whereas HDL levels were significantly lower in patients with NAFLD compared to patients without NAFLD. There was no difference between the groups in other variables.

**Table 1:** Baseline characteristics of the study adult participants by gender with and without NAFLD (n = 2956) Iran, 2016–2017.

Characteristic	Women (n = 1332)					Men (n = 1624)				
	Without NAFLD (n = 725)		With NAFLD (n = 607)			Without NAFLD (n = 871)		With NAFLD (n = 753)		
	N / Mean	% / SD	N / Mean	% / SD	P value	N / Mean	% / SD	N / Mean	% / SD	P value
Age (years)	43.05	15.12	49.56	11.29	< 0.001	48.43	16.31	47.87	13.40	0.45
BMI (kg/m2)	27.53	4.76	32.11	4.72	< 0.001	24.77	3.49	29.19	3.94	< 0.001
Waist circumference (cm)	83.22	10.83	94.33	10.24	< 0.001	84.95	8.82	95.34	9.19	< 0.001
WHtR	0.53	0.07	0.60	0.06	< 0.001	0.50	0.05	0.56	0.05	< 0.001
Smoker, n (%)	4	0.6	3	0.5	0.59	252	28.9	170	22.6	0.002
Alcohol drinker, n (%)	4	0.6	1	0.2	0.24	86	9.9	89	11.8	0.11
Diabetes, n (%)	91	12.6	172	28.3	< 0.001	67	7.7	112	14.9	< 0.001
Metabolic syndrome, n (%)	142	19.6	339	55.8	< 0.001	72	8.3	247	32.8	< 0.001
Heart disease, n (%)	29	4.0	21	3.5	0.35	46	5.3	39	5.2	0.50
Lowering serum glucose agent’s user, n (%)	33	4.7	50	8.8	0.002	32	3.8	54	7.5	0.001
Lowering serum lipid agent’s user, n (%)	81	11.2	108	18.1	< 0.001	82	9.5	80	10.7	0.23
Lowering hypertension agent’s user, n (%)	120	16.6	164	27.0	< 0.001	144	16.5	124	16.5	0.51
Residual areas, n (%)					0.05					0.05
Rural	239	33.0	227	37.4		430	49.84	371	49.3	
Urban	486	67.0	380	62.6		441	50.6	282	50.7	
PA (MET-h/d), n (%)					0.17					0.10
Very low	172	23.9	169	29.0		194	22.4	175	23.3	
Low	124	17.2	92	15.3		92	10.6	106	14.1	
Moderate	259	35.59	224	37.1		325	37.4	250	33.3	
high	166	23.0	118	19.6		257	29.6	219	29.2	
Biochemical parameters										
TG (mg/dl)	108.60	61.05	154.04	110.28	< 0.001	116.82	70.93	163.94	106.49	< 0.001
Total Cholesterol(mg/dl)	179.72	40.81	188.52	42.05	< 0.001	173.32	37.34	183.62	40.25	< 0.001
HDL(mg/dl)	46.79	12.12	45.23	11.45	0.01	42.87	11.47	40.32	11.17	< 0.001
LDL(mg/dl)	98.30	26.46	102.11	26.45	0.009	97.18	26.94	100.43	25.71	0.01
SBP (mmHg)	108.26	18.76	120.11	20.59	< 0.001	113.67	17.39	118.73	18.60	< 0.001
DBP (mmHg)	67.47	11.31	74.62	12.22	< 0.001	70.11	10.47	74.98	11.66	< 0.001
FBS (mg/dl)	101.73	35.25	117.21	44.82	< 0.001	100.18	26.47	107.35	32.30	< 0.001
HbA1C%	4.48	0.90	4.76	0.96	< 0.001	4.45	0.82	4.64	0.96	< 0.001
CRP	1.70	0.70, 4.15	3.00	1.00,6.50	< 0.001	1.00	0.20,2.50	1.80	1.00, 3.50	< 0.001
ALT (mg/dl)	17.52	11.85	22.83	15.71	< 0.001	22.15	13.95	33.53	23.28	< 0.001
AST (mg/dl)	18.72	7.71	20.68	9.03	< 0.001	22.47	12.75	22.47	12.75	0.001
GGT (mg/dl)	22.34	20.59	26.71	16.57	< 0.001	25.25	15.68	25.25	15.68	< 0.001
ALKP (mg/dl)	187.90	70.77	207.51	62.89	< 0.001	197.90	51.34	200.79	52.67	0.26
Dietary parameter										
DED	1.45	0.38	1.48	0.42	0.19	1.56	0.52	1.56	0.46	0.90

*NAFLD* non-alcoholic fatty liver disease, *PA* physical activity, MET: metabolic equivalent of task, *BMI* body mass index, *ALT* Alanine transaminase, *AST* Aspartate transaminase, *GGT* Gamma-glutamyl transferase, *ALKP* Alkaline phosphatase, *CRP* c-reactive protein, *DED* dietary energy density. Data are Mean ± SD (all such values) unless indicated.

Significant at P < .05 for independent t-test for continuous variables and chi-square test for dichotomous variables.

**Table 2:** Components and total scores of HEI-2015 and NRF9.3 according to sex with and without NAFLD among adults participants of Amol Cohort Study (n 2956), Iran, 2016–2017^1^.

Characteristic	Women (n = 1332)					Men (n = 1624)				
	Without NAFLD (n = 725)		With NAFLD (n = 607)		P value	Without NAFLD (n = 871)		With NAFLD (n = 753)		P value
Components of HEI-2015^2,3^										
Adequacy components (score)										
Total fruits (5)	4.42	0.95	4.34	1.05	0.13	4.40	0.97	4.36	0.99	0.42
Whole fruits (5)	4.91	0.36	4.86	0.56	0.03	4.89	0.46	4.90	0.43	0.68
Total vegetables (5)	4.79	0.60	4.76	0.63	0.36	4.73	0.66	4.67	0.73	0.08
Greens and beans (5)	4.23	1.29	4.18	1.31	0.51	4.15	1.31	4.07	1.38	0.22
Whole grains (10)	7.71	3.44	7.85	3.34	0.46	7.61	3.56	7.34	3.62	0.13
Dairy (10)	7.66	2.51	7.76	2.53	0.48	7.25	2.53	7.32	2.51	0.57
Total protein foods (5)	4.07	1.13	4.04	1.17	0.65	4.01	1.16	4.15	1.08	0.01
Seafood and plant proteins (5)	2.42	1.56	2.25	1.47	0.03	2.31	1.54	2.29	1.53	0.84
Fatty acids (10)	6.02	1.69	5.89	1.67	0.15	6.08	1.66	5.83	1.63	0.002
Moderation components (score)										
Refined grains (10)	9.43	1.64	9.39	1.77	0.61	9.44	1.62	9.41	1.68	0.73
Sodium (10)	8.58	2.28	8.33	2.40	0.05	8.57	2.33	8.53	2.31	0.71
Added sugars (10)	8.81	2.23	8.96	2.18	0.23	8.61	2.30	8.53	2.54	0.49
Saturated fats (10)	4.85	3.18	4.55	3.25	0.09	5.70	3.08	5.10	3.06	< 0.001
Total score^4^	76.31	9.34	74.98	10.27	0.01	76.82	10.41	76.19	11.23	0.23
Components of NRF9.3										
Protein (g/d)	1.68	0.33	1.72	0.35	0.06	1.68	0.49	1.72	0.48	0.18
Dietary fiber (g/d)	1.33	0.34	1.32	0.37	0.62	1.31	0.49	1.27	0.34	0.05
Vitamin A (RAE)	0.12	0.08	0.12	0.06	0.03	0.13	0.11	0.12	0.08	0.59
Vitamin C (mg/d)	2.02	1.00	1.85	0.83	0.001	2.04	1.16	1.94	0.86	0.06
Vitamin E (mg/d)	0.51	0.15	0.51	0.14	0.35	0.50	0.30	0.49	0.19	0.52
Calcium (mg/d)	1.12	0.34	1.08	0.31	0.03	1.06	0.36	1.08	0.33	0.30
Iron (mg/d)	1.27	0.64	1.13	0.38	< 0.001	1.25	0.63	1.16	0.44	0.002
Potassium (mg/d)	1.07	0.21	1.05	0.19	0.01	1.05	0.31	1.03	0.18	0.07
Magnesium (mg/d)	0.99	0.17	1.00	0.18	0.48	1.01	0.34	0.99	0.19	0.12
Added sugars (g/d)	0.71	0.63	0.67	0.73	0.26	0.76	0.62	0.81	0.81	0.14
Saturated fats (g/d)	1.35	0.33	1.39	0.37	0.04	1.28	0.42	1.34	0.43	0.001
Sodium (mg/d)	0.93	0.27	1.01	0.52	< 0.001	0.96	0.65	1.00	0.84	0.29
Total score^5^	10.16	2.01	9.81	1.52	< 0.001	10.07	2.89	9.84	1.82	0.05

*NAFLD* non-alcoholic fatty liver disease, *HEI* Healthy Eating Index, *NRF9.3* Nutrient-Rich Food Index 9.3.

Significant at P < .05 for Independent t-test for continuous variables.

^1^Means ± SDs (all such values).

^2^A higher score indicates a higher diet quality, except for added sugars, saturated fats, sodium components., and refined grains.

^3^Maximum score for each component of HEI indicated in parentheses.

^4^Calculated as the sum of all component scores.

^5^Calculated as the sum of scores for nine nutrients to encourage (i.e., protein, dietary fiber, vitamins A, C, and E, calcium, iron, potassium, and magnesium) minus the sum of scores for three nutrients to limit (i.e., added sugar, saturated fats, and sodium).

The scores of nutrient adequacy (p < 0.001) and healthy eating index (p = 0.01) were significantly lower in women with NAFLD than in healthy women. Despite the low score of the healthy eating index and nutritional adequacy in men with NAFLD compared with healthy subjects, these differences were not significant (p > 0.05).

Additional baseline characteristics and dietary assessments across tertile categories of each dietary index (HEI_2015_ and NRF_9.3_) stratified based on gender and NAFLD status are included in Supplementary Tables 1 & 2. A comparison of anthropometric and biochemical parameters across categories of HEI_2015_ showed that in female participants, a higher HEI_2015_ score was associated with lower rates of abdominal obesity (p < 0.001), lower HBA_1c_ (p = 0.04), and a lower intake of dietary energy density (DED) (p < 0.001).

Higher HEI_2015_ and NRF_9.3_ scores were associated with lower diastolic blood pressure among men compared to women (p = 0.02 and p = 0.01, respectively). A less energy-dense diet was also associated with higher HEI_2015_ and NRF_9.3_ scores (p < 0.001) in both genders.

### Association among healthy eating index and nutrient density with NAFLD

The multiple-adjusted odds ratio (95% confidence interval) in Tables 3 and 4 demonstrated an inverse association of nutrient density with NAFLD for the highest (vs. lowest) tertile of NRF_9.3_ [0.68 (0.54–0.85), P _for trend_ = 0.001]. When stratified by gender and abdominal obesity, greater nutrient adequacy was associated with a lower risk of NAFLD in participants with abdominal obesity [0.62 (0.40–0.95), P _for trend_ = 0.03] compared to those without abdominal obesity [0.69 (0.52–0.90), P _for trend_ = 0.007].

**Table 3:** Multiple-adjusted odds ratio and 95% confidence intervals (95% CI) for non-alcoholic fatty liver disease (NAFLD) according to tertiles of dietary index in all adult participants and stratified by sex (n 2956).

	HEI-2015				NRF9.3			
	Tertile 1	Tertile 2	Tertile 3	P_trend_	Tertile 1	Tertile 2	Tertile 3	P_trend_
NAFLD compared with non-NAFLD								
Median score	67.68	75.89	84.36		5.17	6.64	8.43	
NAFLD cases (N, %)	487 (35.8)	472 (34.7)	401(29.5)		442 (32.5)	473 (34.8)	445 (32.7)	
Model 1	ref	0.92 (CI:0.77–1.10)	0.77 (CI:0.64–0.92)	0.005	ref	0.82 (CI:0.68–0.98)	0.80 (CI:0.67–0.97)	0.02
Model 2	ref	0.97 (CI:0.79–1.19)	0.90 (CI:0.73–1.11)	0.34	ref	0.73 (CI:0.59–0.90)	0.71 (CI:0.57–0.88)	0.002
Model 3	ref	1.00 (CI:0.81–1.24)	0.91 (CI:0.73–1.13)	0.40	ref	0.71 (CI:0.57–0.88)	0.68 (CI:0.54–0.85)	0.001
Women								
Median score	68.45	75.84	83.73		5.23	6.61	8.43	
NAFLD cases (N, %)	469 (35.2)	489 (36.7)	374 (28.1)		399 (30.0)	459 (34.5)	474 (35.6)	
Model 1	ref	0.88 (CI:0.68–1.15)	0.81 (CI:0.61–1.08)	0.15	ref	0.76 (CI:0.57–1.00)	0.75 (CI:0.57–0.99)	0.05
Model 2	ref	0.92 (CI:0.68–1.24)	0.97 (CI:0.71–1.34)	0.86	ref	0.71 (CI:0.52–0.97)	0.72 (CI:0.52–0.98)	0.04
Model 3	ref	0.92 (CI:0.67–1.25)	0.91 (CI:0.66–1.27)	0.57	ref	0.66 (CI:0.48–0.92)	0.64 (CI:0.46–0.89)	0.01
Men								
Median score	67.18	75.93	84.71		5.13	6.65	8.13	
NAFLD cases (N, %)	535 (32.9)	522 (32.1)	567 (34.9)		498 (30.7)	594 (36.6)	532 (32.8)	
Model 1	ref	0.97 (CI:0.76–1.23)	0.78 (CI:0.61–0.99)	0.04	ref	0.85 (CI:0.66–1.07)	0.83 (CI:0.64–1.06)	0.13
Model 2	ref	1.02 (CI:0.76–1.36)	0.91 (CI:0.65–1.15)	0.33	ref	0.73 (CI:0.55–0.98)	0.69 (CI:0.51–0.93)	0.01
Model 3	ref	1.06 (CI:0.78–1.43)	0.87 (CI:0.65–1.17)	0.37	ref	0.73 (CI:0.54–0.99)	0.68 (CI:0.50–0.93)	0.01

*HEI* Healthy Eating Index; NRF9.3, Nutrient-Rich Food Index 9.3

Model 1: adjusted for age and sex (except for sex-stratified analysis).

Model 2: Additional adjustment for WC, BMI, energy intake, physical activity, and smoking.

Model 3: additional adjustment for lowering serum lipid drugs, lowering HPTN drugs, lowering serum glucose drugs, residual areas, heart disease, diabetes.

**Table 4:** Multiple-adjusted odds ratio and 95% confidence intervals (95% CI) for non-alcoholic fatty liver disease (NAFLD) according to tertiles of dietary index in all adult participants stratified by waist circumference (n = 2956).

	HEI-2015				NRF9.3			
	Tertile 1	Tertile 2	Tertile 3	P_trend_	Tertile 1	Tertile 2	Tertile 3	P_trend_
NAFLD compared with non-NAFLD Non_abdominal obesity								
NAFLD cases (N, %)	487 (35.8)	472 (34.7)	401 (29.5)		442 (32.5)	473 (34.8)	445 (32.7)	
Model 1	ref	0.99 (CI:0.79–1.24)	0.84 (CI:0.67–1.06)	0.14	ref	0.79 (CI:0.63–0.98)	0.78 CI:0.62–0.98)	0.03
Model 2	ref	1.08 (CI:0.84–1.38)	0.94 (CI:0.73–1.21)	0.65	ref	0.71 (CI:0.55–0.91)	0.70 (CI:0.54–0.90)	0.007
Model 3	ref	1.17 (CI:0.90–1.52)	1.00 (CI:0.77–1.30)	0.97	ref	0.71 (CI:0.55–0.92)	0.69 (CI:0.52–0.90)	0.007
NAFLD compared with non-NAFLD abdominal obesity								
Abdominal obesity^†^ cases	683 (33.0)	683 (33.0)	702 (33.9)		250 (28.2)	318 (35.8)	320 (36.0)	
Model 1	ref	0.77 (CI:0.54–1.08)	0.72 (CI:0.49–1.05)	0.08	ref	0.72 (CI:0.49–1.05)	0.65 (CI:0.45–0.95)	0.03
Model 2	ref	0.77 (CI:0.53–1.10)	0.77 (CI:0.52–1.16)	0.19	ref	0.75 (CI:0.50–1.11)	0.71 (CI:0.47–1.05)	0.10
Model 3	ref	0.69 (CI:0.47–1.01)	0.63 (CI:0.41–0.98)	0.03	ref	0.67 (CI:0.44–1.02)	0.62 (CI:0.40–0.95)	0.03

HEI, Healthy Eating Index; NRF9.3, Nutrient-Rich Food Index 9.3

Model 1: adjusted for age and sex.

Model 2: additional adjustment for BMI, energy intake, physical activity, and smoking.

Model 3: additional adjustment for lowering serum lipid drugs, lowering HPTN drugs, lowering serum glucose drugs, residual areas, heart disease, diabetes.

^†^Abdominal obesity: waist circumference > 102 cm for men and > 88 cm for women.

The results for nutrient quality and lower odds of NAFLD prevalence in both genders were also similar [in men: 0.68 (0.50–0.93), P _for trend_ = 0.01; in women, 0.64 (0.46–0.89), P _for trend_ = 0.01]. The findings of the full multiple-adjusted model of HEI_2015_, stratified by gender and abdominal obesity, showed that the favorable association was only presented on subjects with abdominal obesity [0.63, (0.41–0.98), P _for trend_ = 0.03].

### The link between Nutrient Density and HEI_2015_ with NAFLD and its determinants using path analysis

**Figure 3:**
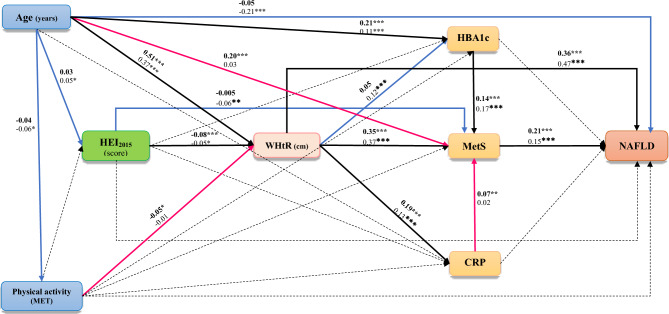
The final path analysis model for the relationship between HEI2015 and NAFLD. The numbers on the paths represent standardized regression coefficients (standardized effects). The bold-face coefficients represent values for women, and the coefficients below them represent values for men. The significance level of the comparison of each effect between men and women is depicted by asterisks (*p < 0.05, **p < 0.01, ***p < 0.001). Pink arrows refer to females whereas blue arrows refer to males. HEI Healthy Eating Index, HBA1c Hemoglobin A1c, MetS Metabolic syndrome, CRP c-reactive protein, NAFLD non-alcoholic fatty liver disease.

The best-fit/final mediation model of HEI_2015_ showed a good-fitting model: χ2/df = 4.08, P = 0.003, GFI = 0.999, AGFI = 0.975, CFI = 0.995, IFI = 0.995, SRMR = 0.015 and RMSEA = 0.032 (Fig. 3).

In both genders, WHtR was a risk factor for CRP (women: standardized β coefficient = 0.19, p < 0.001; men: standardized β coefficient = 0.11, p < 0.001), metabolic syndrome (women: standardized β coefficient = 0.34, p < 0.001; men: standardized β coefficient = 0.36, p < 0.001), and NAFLD (women: standardized β coefficient = 0.36, p < 0.001; men: standardized β coefficient = 0.46, p < 0.001), and was positively associated with HBA_1c_ only in men (standardized β coefficient = 0.11, p < 0.001). Metabolic syndrome in both men and women was a risk factor for NAFLD (women: standardized β coefficient = 0.21, p < 0.001; men: standardized β coefficient = 0.15, p < 0.001). WHtR partially mediated the protective effects of HEI_2015_ on NAFLD (women: standardized β coefficient = –0.08, p < 0.001; men: standardized β coefficient = –0.05, p < 0.001).

The indirect relationship between HEI_2015_ and NAFLD was explained through WHtR, and metabolic syndrome, as well as WHtR, CRP, and metabolic syndrome in women. In men, these effects were partially mediated through WHtR, and metabolic syndrome, as well as WHtR, HBA_1c,_ and metabolic syndrome.

In the final model of NRF_9.3_ in both genders, metabolic syndrome (women: β standardized coefficient = 0.21, p < 0.001; men: standardized β coefficient = 0.25, p < 0.001) was directly related to NAFLD.

WHtR was a risk factor for metabolic syndrome (women: standardized β coefficient = 0.34, p < 0.001; men: standardized β coefficient = 0.36, p < 0.001) and NAFLD (women: standardized β coefficient = 0.36, p < 0.001; men: standardized β coefficient = 0.47, p < 0.001), and there was a direct effect of NRF_9.3_ on WHtR in both genders.

In women, NRF_9.3_ directly (standardized β coefficient = –0.08, p < 0.001) and indirectly through mediating factors of HbA_1c_ (standardized β coefficient = –0.06, p < 0.001), and metabolic syndrome (standardized β coefficient of HBA_1_c to metabolic syndrome = –0.13, p < 0.001) affected NAFLD. In contrast, in men, the protective effect of NRF_9.3_ on NAFLD only was exerted directly (standardized β coefficient = –0.09, p < 0.001).

**Figure 4:**
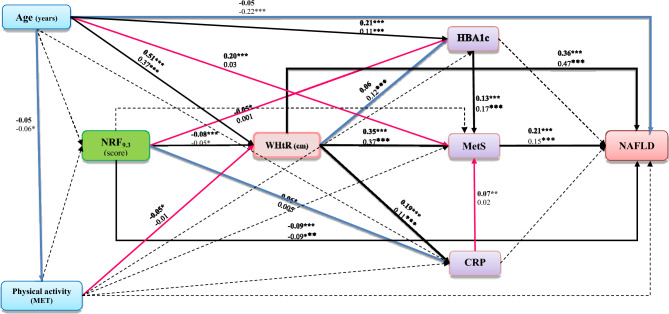
The final path analysis model for the relationship between NRF_9.3_ and NAFLD. The numbers on the paths represent standardized regression coefficients (standardized effects). The bold-face coefficients represent values for women, and the coefficients below them represent values for men. The significance level of the comparison of each effect between men and women is depicted by asterisks (*p < 0.05, **p < 0.01, ***p < 0.001). Pink arrows refer to females whereas blue arrows refer to males. NRF_9.3_, Nutrient-Rich Food Index 9.3, HBA1c Hemoglobin A1c, MetS Metabolic syndrome, CRP c-reactive protein, NAFLD non-alcoholic fatty liver disease.

The best-fit mediation model of NRF_9.3_ showed a well-fitting to the data: χ2/df = 3.97, P = 0.019, GFI = 0.999, AGFI = 0.976, CFI = 0.998, IFI = 0.998, SRMR = 0.012 and RMSEA = 0.032 (Fig. 4).

The standardized path coefficients (β), standardized total effects, along with direct and indirect effects concerning HEI_2015_ and NRF_9.3_ among the participants of the study were documented in Supplementary Table 3.

## Discussion

### Association between nutrient density and diet quality with NAFLD

In the current cross-sectional study, nutrient density (high intake of vitamin A, vitamin C, calcium, potassium, and iron, and the low consumption of sodium and saturated fatty acid) was an independent predictor of reduced prevalence for NAFLD in adult Iranians. The association between diet quality and NAFDL was pronounced in participants with abdominal obesity, where better compliance with the healthy eating index was markedly linked to a lower risk of NAFLD.

Although previous studies have explored the role of individual nutrients in the development, progression, and treatment of NAFLD, nutrient pattern as a total has not been investigated^27,28^.

Total NRF scores (nutrient adequacy) significantly reduced the NAFLD risk by almost 50% in both genders, although there were differences for individual items between patients with NAFLD vs. those without NAFLD. In the current study, low overall intake of nutrients, including vitamin A, vitamin C, potassium, calcium, and iron, and the high consumption of sodium and saturated fatty acid in NAFLD participants, suggest lower the micronutrient density, particularly in women. This pattern was similar only to Iron and SFA among men (Supplementary Table 2).

These findings have been confirmed in other studies^29^. Panera et al. argued that the composition of the macro and micronutrients is more critical in the etiology and management of NAFLD than total calorie intake^30^. Imamura et al., in a systematic assessment of males and females in 187 countries, concluded that females had better dietary patterns than males^31^. However, there is a dearth of evidence indicating gender differences in the association between diet quality and adequacy and NAFLD risk, and the results remain controversial.

According to the HEI components, whole fruit intake was significantly lower in the women with NAFLD than the healthy women, which may suggest that fruit intake reduced NAFLD risk by functioning as a source of vitamin C, vitamin A, calcium, potassium, and dietary fiber^32^. NAFLD is associated with oxidative stress and low-grade inflammation^33^. Micronutrient adequacy protects hepatic cells from lipotoxicity-induced oxidative stress. This status can promote trigger inflammation known to contribute to metabolic dysfunction and disturbing vitamin E trafficking through the gut axis^34^. A higher intake of vitamin C, vitamin A, calcium, and potassium is a major preventive nutrient for metabolic syndrome and NAFLD^35^. Van Tien et al., in a Multi-institutional Collaborative Cohort of the Japanese population using 1588 subjects, proposed that a nutrient diet rich in vitamins, fiber, iron, and potassium was linked to a lower prevalence of NAFLD^36^. A cohort study assessing dietary intake in NAFLD patients found that recommended intakes of calcium, vitamin A, iron, vitamin B1, vitamin B2, zinc, and magnesium were not met^37^. Aktary et al.^38^, in a cross-sectional investigation of the dietary intake and health profile of a sample of Canadian adults with NAFLD (n = 42), demonstrated that NAFLD patients had poor micronutrients such as magnesium, calcium, vitamin D, and vitamin E. The increased consumption of saturated fatty acid has been shown to cause mitochondrial dysfunction, increased oxidative stress, and low-grade inflammation^39^. Moreover, a recent meta-analysis documented a positive association between high sodium consumption and a 60% greater risk of NAFLD^40^.

In both genders, NAFLD patients significantly had a lower intake of iron and higher consumption of saturated fatty acids than healthy participants did. The association between select micronutrients and NAFDL warrants further investigation.

The association between diet quality and NAFLD aligned with those in a US population-based study (n = 10,858, mean age = 42.9 years, 47.1% men)^40^. Women (healthy vs. NAFDL participants) had more consumption of seafood, plant proteins, and whole fruits, while healthy men consumed better fatty acid composition (greater ratio of polyunsaturated (PUFAs) and monounsaturated fatty acids (MUFAs) to SFA) relative to NAFLD participants.

The reason the relationship was significant only among those with abdominal obesity might be that these participants were more prone to have NAFLD^41^, and weight loss as primary therapy for most NAFLD patients has been documented^42^. Improving diet quality, for example, incorporating more vegetables, whole fruits, whole grains, and limiting the content of the SFA sources, may have important benefits in preventing weight gain or promoting weight loss in adults of both genders and even being predisposed to obesity^43,44^. Thus, implementing healthy dietary patterns in the clinical setting may be a viable alternative to encourage weight loss and reduce the risk of developing NAFLD; however, more research is required to identify short-term and long-term beneficial effects of these dietary interventions in the context of NAFLD.

Considering these findings, primary prevention of NAFLD by improving micronutrient adequacy may be a more effective and beneficial objective for the patient at risk of developing NAFLD.

### Influencing factors of NAFLD

Based on the gender-stratified path analysis, in women, diet quality indirectly through WHtR, CRP, and metabolic syndrome, and in men through WHtR, Hemoglobin A_1c_, and metabolic syndrome affected NAFLD.

While nutrient density directly and indirectly (through HBA_1c_ and metabolic syndrome) reduced NAFLD risk in women, it only had a direct protective effect on NAFLD in men.

The current findings apply to understanding how habitual diet shapes anthropometric indices, metabolic risk profiles, and health outcomes in the Iranian surveyed population.

The findings indicate age is a significant predictor of HEI, physical activity, cardiometabolic parameters (e.g., WHtR, HBA1c), and NAFLD risk. Previous studies revealed differences in lifestyle factors (e.g., eating behaviors and physical activity) based on age and gender^45^. Age directly influences diet quality, and is a significant predictor of physical activity^46^. Research findings affirmed that increased physical activity was correlated with better diet quality measured by HEI 2015^47,48^.

NAFLD risk has been more prevalent in older people^49^. Asian studies also reported that under the age of 50 years, NAFLD was more prevalent in men, but in populations over 50 years, it was higher in women^50,51^. Processes associated with aging are considered possible contributing mechanisms in the pathogenesis of NAFLD and cardiometabolic disorders^52^.

In line with our results, growing evidence demonstrated obesity and metabolic syndrome were independently linked with NAFLD irrespective of other cardiometabolic risk factors^53–55^. The NAFLD's pathophysiology concerning obesity involves excess fat deposition in the liver and insulin resistance development, which are pivotal in the progression of NAFLD^56^. In a recent study, the correlation between cardiometabolic disorders and inflammation and the incidence of NAFLD was verified^57^.

Evaluating overall diet quality, rather than specific nutrients or food components, is more effective in identifying diet-disease associations^5^. HEI is an indicator of determining the nutritional balance and predicting health risks. However, there are discrepancies in the risk prediction of disease in diet quality because of unmeasured interactions with various effects of modifiers or mediators^41^. Furthermore, the relationship and interrelationship between diet quality and health risk cannot be precisely calculated using common statistical methods. Path analysis may help evaluate this connection within a conceptual framework by concurrently investigating all relevant regression pathways, including direct and indirect^7^. Applying Path analysis makes it easier to assess the mediating role of diet quality and adequacy, anthropometric and metabolic parameters, and NAFLD risk. Additionally, this method permits a thorough understanding of such a relationship and allows a more precise interpretation of results.

A higher HEI score signifies a more balanced nutritional intake, leading to improved insulin sensitivity, reduced inflammatory markers, and a decreased likelihood of metabolic syndrome^58^. The NRF index, unlike the HEI, evaluates individual foods and simultaneously provides a precise measure of overall diet nutrient density. Individuals can meet their nutrient requirements without excessive energy intake and gaining weight by choosing nutrient-dense foods^59^. Moreover, the NRF index prioritizes nutrients critical for preventing metabolic disorders and NAFLD^60,61^.

Although the rationale for WHtR, CRP, and HBA_1c_ as strong predictors for NAFLD has been justified by several previous studies^40^, to our knowledge there is no research on metabolic dysfunction being a mediator of NAFLD as the present study is the only Path analysis modeling study in this regard. A meta-analysis reported the superiority of centralized obesity measures, particularly, WHtR, for NAFLD risk detection^62^. Evidence has shown that visceral adiposity is the main adipose depot responsible for fatty liver and is associated with it in a dose-dependent manner^37^. In the present study, high WHtR values were associated with CRP and HBA_1c_. Several epidemiologic studies have proved the causal link between obesity and increasing liver disease in individuals^63^. According to pathophysiology and clinical studies, the progression of NAFLD is caused by an imbalance between lipid intake and disposal, which leads to oxidative stress and hepatocyte injury^63^. This finding is important to broaden the discussion about the high level of early inflammatory markers in obese adults and clarify this relationship. CRP acts as a regulator of nitric oxide production in the endothelium and coordinates the production and secretion of various cytokines, increasing the pro-inflammatory activity of different adipokines. The measurement of CRP and HBA_1c_ were independent predictors of metabolic syndrome in other cohort studies (OR 1.22, 95% CI; 1.12 to 1.35; OR 1.57, 95% CI; 1.35 to 1. 82, respectively)^6,8^. MetS was associated with oxidative stress and chronic low-grade inflammation^63^, and NAFLD is one criterion of MetS^30^.

Additionally, our results revealed a gender inequalities association of diet quality and adequacy with anthropometric and metabolic parameters and NAFLD. The beneficial effects of nutrient adequacy of NAFLD risk in women, directly and indirectly through changes in HBA1c and metabolic syndrome, and in men directly were potentially exerted.

These results reveal the importance of gender-specific interventions to control NAFLD and also the pivotal role of diet adequacy and adequacy in obesity and glycemic control and preventing low-grade systemic inflammation and metabolic syndrome among high-risk individuals.

Key strengths of the study include the relatively large community-based study sample recruited from rural and urban areas of Amol city that afforded us sufficient power to probe small effects, incorporating multiple potential anthropometric and biochemical mediators, and assessing their mediation role simultaneously in the relationship between diet quality and adequacy and the risk of NAFLD for the first time, using a reliable and validated semi-quantitative FFQ^64^ developed for the Iranian population, which results in a better representation of the participants' dietary habits. However, some potential limitations of this study need to be acknowledged. First, because of the cross-sectional design of the study, drawing any causal inference from the association would be incorrect. Second, although liver biopsy is a gold standard for diagnosing NAFLD, we used sonography for evaluating NAFLD due to the risks associated with liver biopsy and the impossibility of applying it in population-based studies. Furthermore, the sensitivity of the ultrasound for the detection of moderate to severe fatty liver is approximately 85%, which keeps it a preferred and practical modality for diagnosing NAFLD in epidemiological settings. Third, since dietary intake and other socio-demographic parameters in Amol may differ from those in other parts of the country, our results cannot be extended to all Iranians. Fourth, other effective factors, including meal and snack patterns and cooking methods, were not investigated in the current study, so the observed associations are not entirely explained. Finally, we could not completely rule out residual confounding due to unknown or unmeasured confounders in this study.

## Conclusion

Nutrient density was an independent predictor of NAFLD prevalence in Iranian adults. The association between diet quality (assessed by the HEI_2015_) and NAFDL was more pronounced in participants with abdominal obesity. The beneficial indirect effects of diet quality and nutrient density on NAFLD prevention were mediated by changing WHtR, HBA1c, CRP, and metabolic syndrome. Therefore, for subjects with MetS, high WHtR, high HBA1c, and CRP, we can provide early dietary intervention and proper education to prevent progression to NAFLD. Future research assessing the longitudinal relationship using prospective study designs is needed to better understand these relationships and confirm the findings in the present study.

## Methods

### Study design, setting, and participants

This cross-sectional study was conducted within the framework of the Amol Cohort Study (AmolCS), a prospective study conducted on rural and urban residents of Amol City in the North of Iran, which evaluated obesity-related metabolic disorders and CVD. The AmolCS was set up in two phases. In the first phase started in 2009, 7104 participants aged 10–90 years through sixteen strata with ten-year intervals (10–19, 20–29, 30–39, 40–49, 50–59, 60–69, 70–79, and 80–89 years) were randomly selected across rural and urban health centers of Amol city. The second phase of the AmolCS, including 5147 adult participants ≥ 18 years of age, was launched in 2017, and the data from the second phase of the cohort study were used in the present analysis.

The exclusion criteria for the participants were pregnancy/lactation, following a specific dietary or physical activity regimen, history of disease including Wilson's disease, autoimmune liver disease, hemochromatosis, virus infection, alcoholic fatty liver, malignancy, thyroid disorder, and autoimmune diseases, as well as participants with significant alcohol consumption (> 30 g/d for men and > 20 g/d for women). Written informed consent was obtained from all participants before the study. Further details on the project are available in the previous studies^65,66^.

In total, 2956 subjects, including 1332 women (45.1%) and 1624 men (54.9%), were evaluated after excluding missing data for the abdominal ultrasonography (n = 166), covariates (n = 186), the food frequency questionnaire (n = 249), and misreported energy intake values (n = 492).

The study design and selection flowchart are outlined in Fig. 2. Approval for this study was received from the Iran University of Medical Sciences (IUMS) ethics committee (NO: IR.IUMS.REC.1399.1393).

### Data collection

Written and verbal informed consent was obtained from participants. The documentation of participants' information in the second phase of the cohort study included demographic and lifestyle characteristics, clinical testing results, dietary assessment, and NAFLD diagnosis.

### Dietary assessment

A validated semi-quantitative food-frequency questionnaire (FFQ) was used to evaluate the habitual intake of 168 food items^64^. For each food item on the list, participants were asked about the usual frequency of consumption in a commonly used unit or portion size (daily, weekly, and monthly) over the previous year. The consumption intake of each food item was calculated as grams/day by household measures^67^. Nutrient and energy intake was calculated using the food composition table (FCT) of the United States Department of Agriculture (USDA)^68^ and the Iranian FCT for traditional Iranian foodstuffs^69^.

#### Healthy eating index and nutrient density

In order to evaluate the quality of the diet, the Healthy Eating Index 2015 (HEI_2015_) was calculated using the method explained by the National Cancer Institute and the US Department of Agriculture (USDA) center^70,71^. In this index, nutritional intakes were compared with the US dietary guidelines. Scores could range between 0 and 100, with a higher score suggesting a healthier diet. The Nutrient Rich Food 9.3 score (NRF_9.3_) was calculated for the whole diet to measure nutrient density. Drewnowski et al.^72^ described the details of NRF_9.3_ calculation. In brief, the calculation of NRF9.3 is based on nine qualifying nutrients, including protein, fiber, vitamins A, C, and D, calcium, magnesium, potassium, and iron; and three disqualifying nutrients, including saturated fat, added sugar, and sodium. NRF_9.3_ was calculated as the sum of the percentage of the reference daily values (RDVs) for qualifying nutrients (NR_9_) minus the sum of the percentage of maximum recommended value (MRVs) for disqualifying nutrients (Lim_3_). All daily values calculated per 2000 kcal and the RDVs and MRVs suggested by Drewnowski et al. al. (based on several sources, i.e., WHO and FDA)^72^ were used in the present study.

### Diagnosis and assessment of NAFLD

All the study subjects underwent ultrasonography of the abdomen to assess the hepatic parenchyma and biliary tree, performed by a single expert radiologist blinded to the clinical, and laboratory data of the participants using an ultrasound system (Esaote SpA, Genova, Italy) with transducer (frequency bandwidth 3–5 MHZ).

### Laboratory testing

After an overnight fast of at least 12 h, intravenous blood samples of each participant were collected, with one tube for Ethylene Diamine Tetraacetic Acid (EDTA) anticoagulation and one tube for separation gel coagulation, and then centrifuged at 3000 rpm for 10 min at 4 °C; the aliquots were stored at -80 °C until use. Fasting blood sugar (FBS) was measured by the hexokinase method, and lipid profile containing total cholesterol (TC), high-density lipoprotein cholesterol (HDLc), and low-density lipoprotein cholesterol (LDLc) were measured by the enzymatic method using an Auto-analyzer BS200 (Mindray, Shenzhen, China) and diagnostic kits (Pars Azmoon Co., Tehran, Iran). Alanine transaminase (ALT), aspartate transaminase (AST), γ-glutamyl transaminase (GGT), and CRP were measured with a rating method.

Hepatitis B surface antibody, hepatitis B surface antigen, hepatitis C virus antibody, and hepatitis B core antibody were assessed by Enzyme-linked immunosorbent assay (ELIZA) kits (Pishtaz Teb Co., Tehran, Iran). Ten percent of the blood samples were re-evaluated by the Iranian National Reference Laboratory. The coefficients of variations ranged from 1.7% to 3.8% for all laboratory values.

### Anthropometric variables and covariates

For each participant, covariates of demographic and lifestyle characteristics, smoking and alcohol drinking status, and physical activity were collected with the questionnaire-based interview. Data on physical activity was completed using the validated international physical activity questionnaire (IPAQ), as metabolic equivalent minutes per minute per week (MET-min/week)^73^.

Trained assistants employed medical equipment to measure height (cm), weight (kg), waist circumference (WC, cm), systolic blood pressure (SBP), and diastolic blood pressure (DBP). Height and weight were measured with the subjects wearing light clothing and no shoes. Height was recorded at the nearest 0.1 cm and weight was to the nearest 0.1 kg. Body mass index (BMI) was computed as weight (kg) divided by height squared (m^2^). The girth of the midpoint between the lowest point of the rib and the upper edge of the iliac crest was calculated as waist circumference (WC). The WC measurement was taken to the nearest 0.1 cm. WHtR was respectively calculated as WC divided by height^74^.

Blood pressure was measured 2 times after at least 5 min of rest using the standardized desktop sphygmomanometer. The average blood pressure derived from two measurement readings was used^75^. All variables were collected according to standard interview guidelines and standard protocols^75,76^.

### Data analysis

#### Descriptive analysis

Descriptive statistics included the frequency count (percent) for categorical variables and mean and standard deviation (SD) for continuous variables. The normality of continuous variables was evaluated by the Shapiro–Wilk statistical test. Baseline characteristics and dietary intake of the participants across tertile categories of each dietary index (HEI_2015_ and NRF_9.3_) were compared by conducting a one-way analysis of variance (ANOVA) with a Bonferroni post-hoc analysis to make multiple comparisons for continuous variables and chi-square test for categorical variables. The associations were adjusted for energy intake.

#### Logistic regression

To examine the association between nutrient density and healthy eating indices with NAFLD, multiple logistic regression was used in several models for all participants. The obtained findings were adjusted for confounding factors, including age, sex, WC, BMI, energy intake, physical activity, and smoking. Further adjustment for lowering serum lipid drugs, lowering hypertension (HPTN) drugs, lowering serum glucose drugs, residual areas, the presence of heart disease, and diabetes was applied in the last model. Stratified analyses by gender, as well as waist circumference status, were also conducted. We used tertile categories as an ordinal variable to assess the trend of odds ratios across increasing tertiles of dietary indxes scores. For potential confounding factors, a univariate analysis was applied, and those with a P-value for entry (Pe) lower than 0.20 were included in the final multiple models.

#### Path analysis model

A path analysis model was utilized to assess the hypothesized model. Path analysis is based on the maximum-likelihood estimation of the entire system of the hypothesized model and assesses the degree to which the data fits the specified model^77^. In the present analysis, we performed a two-step strategy outlined by Anderson and Gerbing^78^. The initial hypothesized model was evaluated by Path analysis to measure the fit and path coefficients. We computed the standardized regression weights, standardized total effects, as well as direct and indirect effects.

The goodness-of-fit indices of models and their corresponding suggested thresholds were: Goodness-of-fit Index (GFI) > 0.90, adjusted goodness-of-fit index (AGFI) > 0.90, comparative fit index (CFI) > 0.90, incremental fit index (IFI) ≥ 0.9, root-mean-square error of approximation (RMSEA) ≤ 0.08, and standardized root mean square residual (SRMR) < 0.08^79,80^.

In this study, the significance level was set at 5%, and all reported P-values are based on two-sided tests and the corresponding 95% confidence interval (CI). All the statistical analyses were done via SPSS version 24 (Statistical Package for Social Science, SPSS Inc, Chicago, IL, USA) software. A path analysis model was employed using AMOS 23.0 to build a measurement model and verify the structural relationship between nutrient density and healthy eating index with NAFLD.

### Ethics statement

The current study was conducted according to the guidelines in the Declaration of Helsinki, and procedures involving human subjects/patients were approved by the Iran University of Medical Sciences (IUMS) ethical committee (No.IR.IUMS.REC.1399.1393). Written informed consent was obtained from all participants before the study.

### Supplementary Information

Supplementary Information.

Supplementary Table S1.

Supplementary Table S2.

Supplementary Table S3.

## Data Availability

The datasets used and/or analyzed during the current study are available from the corresponding author upon reasonable request. Interested researchers may contact the corresponding author, Prof. Farhad Zamani, email address: zamani.farhad@gmail.com.

## Abbreviations

ADG: American Dietary Guideline
AGFI: Adjusted goodness-of-fit index
ALT: Alanine transaminase
AST: Aspartate transaminase
BMI: Body mass index
CFI: Comparative fit index
CRP: C-reactive protein
DBP: Diastolic blood pressure
FBS: Fasting blood sugar
FCT: Food composition table
FFQ: Food-frequency questionnaire
GFI: Goodness-of-fit Index
GGT: γ-Glutamyl transaminase
HBA1c: Hemoglobin A_1c_
HDLc: High-density lipoprotein cholesterol
HEI_2015_: Healthy Eating Index 2015
IFI: Incremental fit index
IPAQ: International physical activity questionnaire
LDLc: Low-density lipoprotein cholesterol
MetS: Metabolic syndrome
MRVs: Maximum recommended value
NRF_9.3_: NuFtrient-Rich Food 9.3
MAFLD: Metabolic-associated fatty liver disease
NAFLD: Nonalcoholic Fatty liver disease
RDVs: Reference daily values
RMSEA: Root-mean-square error of approximation
SBP: Systolic blood pressure
SD: Standard deviation
SRMR: Standardized root mean square residual
TC: Total cholesterol
USDA: US Department of Agriculture
WC: Waist circumference
WHtR: Waist-to-height ratio
