# Supplementary material for: Path analysis model to identify the effect of poor diet quality on NAFLD among Iranian adults from Amol Cohort Study
Source: Sci Rep. 2024 Aug 27;14:19935. doi: 10.1038/s41598-024-70181-4 (PMC11358441; doi:10.1038/s41598-024-70181-4)
Supplement: Supplementary file 1 — Supplementary Information. [file 41598_2024_70181_MOESM1_ESM.docx]

Supplementary Info:

**Path analysis model to identify the effect of poor diet quality on NAFLD among Iranian adults from Amol Cohort Study**

Azam Doustmohammadian^1^, Bahareh Amirkalali ^1^, Barbora de Courten ^2^, Saeed Esfandyari ^3^, Nima Motamed^4^, Mansooreh Maadi^1^, Hossein Ajdarkosh^1^, Esmaeel Gholizadeh^1^, Samira Chaibakhsh^5^, Farhad Zamani^1^*

^1^ Gastrointestinal and Liver Diseases Research Center, Iran University of Medical Sciences, Tehran, Iran

^2^ School of Health and Biomedical Sciences, RMIT University, Melbourne, VIC 3085, Australia

^3^Asadabad School of Medical Sciences, Hamadan, Iran

^4^ Department of Social Medicine, Zanjan University of Medical Sciences, Zanjan, Iran

^5^ Echocardiography Research Center, Rajaie Cardiovascular Medical and Research Center, Iran University of Medical Sciences, Tehran, Iran

*Correspondence to: Farhad Zamani

**Literature-based schematic model**

Figure 1 depicts a schematic pathway comprising baseline predictors, mediators, and the NAFLD outcome. According to the literature review, the following hypotheses were tested:

**Hypothesis 1.1 (H_1.1_)**: Demographic factors are associated with lifestyle factors, including dietary index (NRF and HEI), and physical activity.

Age-and gender-related differences have been reported in relation to lifestyle factors in the previous studies ^1^. According to the results, women usually have higher HEI and NRF ^2^, age is directly associated with the HEI_2015_ total score ^2^, and both are significant predictors of physical activity and HEI scores ^3^.

**Hypothesis 1.2 (H_1.2_):** Demographic factors are associated with the waist-to-height ratio (WHtR).

Some demographic factors, such as older age and male gender, were associated with an increased risk of abdominal obesity defined as WHtR ^4^.

**Hypothesis 1.3 (H_1.3_):** Demographic factors are associated with the c-reactive protein (CRP).

The finding of demographic differential of CRP research shows that age and male gender are independently associated with CRP concentration in East Asia people ^5^.

**Hypothesis 1.4 (H_1.4_):** Demographic factors are associated with hemoglobin A_1c_ (HBA1c).

Population-based cohort studies have pointed to a significant association between age and male gender and glucose levels in healthy people ^6,7^.

**Hypothesis 1.5 (H_1.5_):** Demographic factors are associated with metabolic syndrome (MetS).

The effect of demographic characteristics, including age, on the prevalence of MetS, has been stated in many studies ^8,9^. The interaction between age and sex on MetS has been significant, and the risk of MetS has increased in the ages of 50-70 years in both genders ^10^.

**Hypothesis 1.6 (H_1.6_):** Demographic factors are associated with NAFLD.

Age and gender also affect the prevalence of NAFLD. The disease has been more prevalent in older people and women ^11^. Asian studies also report that under the age of 50 years, NAFLD is more prevalent in men, but in populations over 50 years, it is higher in women ^12^.

**Hypothesis 2.1 (H_2.1_):** Lifestyle factors (e.g., physical activity) are associated with improving NRF and HEI.

Studies have shown that increasing the duration of physical activity is related to reducing the consumption of unhealthy foods ^13^. Among college students, better physical activity levels are also linked to better diet quality, measured by HEI_2015_ ^14^.

**Hypothesis 2.2 (H_2.2_):** Lifestyle factors are in association with the WHtR.

The findings suggest that lifestyle modification including physical exercise is important in the prevention of obesity as well as long-term weight maintenance ^15^.

**Hypothesis 2.3 (H_2.3_):** Lifestyle factors are associated with inflammatory biomarkers.

Meta-analyzes results showed the effectiveness of physical exercise in reducing the inflammatory markers ^16^

**Hypothesis 2.4 (H_2.4_):** Physical activity is associated with HBA1c. It is widely believed that engaging in physical activity can help individuals with type 2 diabetes manage their glucose levels and prevent the development of the disease. Further, exercise-based interventions have been shown to reduce glucose levels in healthy people ^17^.

**Hypothesis 2.5 (H_2.5_):** Lifestyle factors are associated with MetS.

Diet and physical activity are the key factors affecting the prevalence of MetS ^18^.

**Hypothesis 2.6 (H_2.6_):** Lifestyle factors are in association with NAFLD.

Different studies have shown the effect of lifestyle modification, such as diet and exercise, on improving NAFLD ^19^.

**Hypothesis 3.1 (H_3.1_)**: NRF and HEI are associated with WHtR.

WHtR can be effectively used to assess the effect of lifestyle on obesity and there has been a positive association between WHtR and HEI ^20^.

**Hypothesis 3.2 (H_3.2_):** NRF and HEI are associated with inflammatory biomarkers.

There is an agreement that healthy dietary patterns are inversely associated with the level of inflammatory biomarkers ^21^. Dietary patterns low in red meat, sugary drinks, processed foods, and saturated fat and high in vegetables, green leaves, fruits, whole grain cereals, chestnuts, fish, and olive oil, are usually associated with a lower inflammatory state ^22^.

**Hypothesis 3.3 (H_3.3_):** NRF and HEI are associated negatively with HbA1c, insulin resistance, and type 2 diabetes ^23,24^.

**Hypothesis 3.4 (H_3.4_):** NRF and HEI are associated with metabolic syndrome.

HEI has revealed a reverse linear association with BMI and other components of metabolic syndrome such as systolic and diastolic blood pressure, serum TG, low-density lipoprotein, and total cholesterol level ^25,26^. Scientific results indicate a better diet quality, assessed by HEI or adherence to healthy dietary patterns (such as DASH, Mediterranean diet), reduces the risk of MetS ^27^.

**Hypothesis 3.5 (H_3.5_):** NRF and HEI are associated with NAFLD.

Better diet quality assessed by different indexes, including HEI, is associated with a lower risk of NAFLD ^28^.

**Hypothesis 4.1 (H_4.1_):** WHtR is associated with inflammatory biomarkers.

Based on epidemiologic studies, WHtR is the best predictor for inflammation markers, including CRP, interleukin-6 (IL-6), and homocysteine ^29,30^.

**Hypothesis 4.2 (H_4.2_):** WHtR is associated with HbA1c.

Studies report a strong association of WHtR with insulin resistance, prediabetes, and type 2 diabetes ^31,32^.

**Hypothesis 4.3 (H_4.3_):** WHtR is associated with MetS.

Previous studies indicate that the WHtR is superior to other obesity indices, such as BMI or waist circumference in predicting MetS, especially in adults without obesity ^33^.

**Hypothesis 4.4 (H_4.4_):** WHtR is the best predictor of NAFLD ^34^.

**Hypothesis 5.1 (H_5.1_):** Inflammatory biomarkers are associated with MetS.

Inflammatory biomarkers are associated with the incidence of metabolic syndrome, and it is suggested that CRP and IL-6 can be used in assessing the risk of metabolic syndrome ^35^.

**Hypothesis 5.2 (H_5.2_):** Inflammatory biomarkers are associated with NAFLD.

Previous studies indicate that higher levels of inflammatory biomarkers, including CRP, IL-1β, IL-6, and TNF-α, are associated with an increased risk of NAFLD and its higher grade. These biomarkers can have an important role in the etiology of NAFLD ^36^.

**Hypothesis 6.1 (H_6.1_):** HbA1c is associated with MetS.

According to the studies, the HbA1c level, which shows the average blood sugar in the last three months, is better than fasting blood sugar in the prediction of metabolic syndrome ^19,37^.

**Hypothesis 6.2 (H_6.2_):** HbA1c is associated with NAFLD.

The results of several studies have shown that independent of obesity and other metabolic disorders, the increase in HbA1c levels is related to the increase in the incidence of NAFLD and its progression, especially in individuals without diabetes ^21,38^.

**Hypothesis 7 (H_7_):** MetS is associated with NAFLD.

Various studies have reported the link between metabolic syndrome and the progression of NAFLD ^39^.

**References**

1 Kang, M. *et al.* Sex differences in sociodemographic and lifestyle factors associated with diet quality in a multiethnic population. *Nutrition* **66**, 147-152 (2019). <https://doi.org/10.1016/j.nut.2018.11.022>

2 Shah, B. S., Freeland-Graves, J. H., Cahill, J. M., Lu, H. & Graves, G. R. Diet quality as measured by the healthy eating index and the association with lipid profile in low-income women in early postpartum. *Journal of the American Dietetic Association* **110**, 274-279 (2010). <https://doi.org/10.1016/j.jada.2009.10.038>

3 Sutherland, L. A., Kaley, L. A. & Fischer, L. Guiding stars: the effect of a nutrition navigation program on consumer purchases at the supermarket. *Am J Clin Nutr* **91**, 1090s-1094s, doi:10.3945/ajcn.2010.28450C (2010). <https://doi.org/10.3945/ajcn.2010.28450C>

4 Setiono, F. J., Guerra, L. A., Leung, C. & Leak, T. M. Sociodemographic characteristics are associated with prevalence of high-risk waist circumference and high-risk waist-to-height ratio in US adolescents. *BMC pediatrics* **21**, 215 (2021). <https://doi.org/10.1186/s12887-021-02685-1>

5 Yu, Y., Cai, J., She, Z. & Li, H. Insights into the epidemiology, pathogenesis, and therapeutics of nonalcoholic fatty liver diseases. *Advanced Science* **6**, 1801585 (2019). <https://doi.org/10.1002/advs.201801585>

6 George, E. S. *et al.* Exploring the Path of Mediterranean Diet, Non-Alcoholic Fatty Liver Disease (NAFLD) and Inflammation towards 10-Year Cardiovascular Disease (CVD) Risk: The ATTICA Study 10-Year Follow-Up (2002–2012). *Nutrients* **14**, 2367 (2022). <https://doi.org/10.3390/nu14122367>

7 Mellergård, E., Johnsson, P. & Eek, F. Sociodemographic factors associated with HbA1c variability in type 2 diabetes: a prospective exploratory cohort study. *BMC Endocrine Disorders* **20**, 1-8 (2020). <https://doi.org/10.1186/s12902-020-00585-6>

8 Chan, R. *et al.* Diet-quality scores and prevalence of nonalcoholic fatty liver disease: a population study using proton-magnetic resonance spectroscopy. *PLoS One* **10**, e0139310 (2015). <https://doi.org/10.1371/journal.pone.0139310>

9 Olza, J. *et al.* Waist-to-height ratio, inflammation and CVD risk in obese children. *Public health nutrition* **17**, 2378-2385 (2014). <https://doi.org/10.1017/S1368980013003285>

10 Loomba, R. & Sanyal, A. J. The global NAFLD epidemic. *Nature reviews Gastroenterology & hepatology* **10**, 686-690 (2013). <https://doi.org/10.1038/nrgastro.2013.171>

11 Jamar, G. *et al.* Evaluation of waist-to-height ratio as a predictor of insulin resistance in non-diabetic obese individuals. A cross-sectional study. *Sao Paulo Medical Journal* **135**, 462-468 (2017). [ttps://doi.org/10.1590/1516-3180.2016.0358280417](https://doi.org/10.1590/1516-3180.2016.0358280417)

12 Yang, H., Xin, Z., Feng, J.-P. & Yang, J.-K. Waist-to-height ratio is better than body mass index and waist circumference as a screening criterion for metabolic syndrome in Han Chinese adults. *Medicine* **96** (2017). <https://doi.org/10.1097/MD.0000000000008192>

13 Zeng, J. *et al.* Prevalence, clinical characteristics, risk factors, and indicators for lean Chinese adults with nonalcoholic fatty liver disease. *World journal of gastroenterology* **26**, 1792 (2020). <https://doi.org/10.3748/wjg.v26.i15.1792>

14 Xu, F. *et al.* Relationships of physical activity and diet quality with body composition and fat distribution in US adults. *Obesity* **28**, 2431-2440 (2020). <https://doi.org/10.1002/oby.23018>

15 Swift, D. L. *et al.* The effects of exercise and physical activity on weight loss and maintenance. *Progress in cardiovascular diseases* **61**, 206-213 (2018). <https://doi.org/10.1016/j.pcad.2018.07.014>

16 Xian, Y.-X., Weng, J.-P. & Xu, F. MAFLD vs. NAFLD: shared features and potential changes in epidemiology, pathophysiology, diagnosis, and pharmacotherapy. *Chinese Medical Journal* **134**, 8-19 (2021). <https://doi.org/10.1097/CM9.0000000000001263>

17 Bashir, A., Duseja, A., De, A., Mehta, M. & Tiwari, P. Non-alcoholic fatty liver disease development: A multifactorial pathogenic phenomena. *Liver Research* (2022). <https://doi.org/10.1016/j.livres.2022.05.002>

18 Kraja, A. T. *et al.* Do inflammation and procoagulation biomarkers contribute to the metabolic syndrome cluster? *Nutrition & metabolism* **4**, 1-12 (2007). <https://doi.org/10.1186/1743-7075-4-28>

19 Ong, K. L. *et al.* Using glycosylated hemoglobin to define the metabolic syndrome in United States adults. *Diabetes Care* **33**, 1856-1858 (2010). <https://doi.org/10.2337/dc10-0190>

20 Park, S., Barrett-Connor, E., Wingard, D. L., Shan, J. & Edelstein, S. GHb is a better predictor of cardiovascular disease than fasting or postchallenge plasma glucose in women without diabetes: the Rancho Bernardo Study. *Diabetes care* **19**, 450-456 (1996). <https://doi.org/10.2337/diacare.19.5.450>

21 Fan, J. G. *et al.* Guidelines for the diagnosis and management of nonalcoholic fatty liver disease: update 2010:(published in Chinese on Chinese Journal of Hepatology 2010; 18: 163-166). *Journal of digestive diseases* **12**, 38-44 (2011). <https://doi.org/10.1111/j.1751-2980.2010.00476.x>

22 Chobanian, A. V. *et al.* Seventh report of the joint national committee on prevention, detection, evaluation, and treatment of high blood pressure. *hypertension* **42**, 1206-1252 (2003). <https://doi.org/10.1161/01.HYP.0000107251.49515.c2>

23 World Health Organization. Waist circumference and waist-hip ratio: report of a WHO expert consultation, Geneva, 8-11 December 2008. (2011).

24 Chiuve, S. E. *et al.* Alternative dietary indices both strongly predict risk of chronic disease. *The Journal of nutrition* **142**, 1009-1018 (2012). <https://doi.org/10.3945/jn.111.157222>

25 Mouzaki, M. & Allard, J. P. The role of nutrients in the development, progression, and treatment of nonalcoholic fatty liver disease. *Journal of clinical gastroenterology* **46**, 457-467 (2012). <https://doi.org/10.1097/MCG.0b013e31824cf51e>

26 Tan, S.-Y., Georgousopoulou, E. N., Cardoso, B. R., Daly, R. M. & George, E. S. Associations between nut intake, cognitive function and non‐alcoholic fatty liver disease (NAFLD) in older adults in the United States: NHANES 2011-14. *BMC geriatrics* **21**, 1-12 (2021). <https://doi.org/10.1186/s12877-021-02239-1>

27 Imamura, F. *et al.* Dietary quality among men and women in 187 countries in 1990 and 2010: a systematic assessment. *The lancet global health* **3**, e132-e142 (2015). <https://doi.org/10.1016/S2214-109X(14)70381-X>

28 Kim, D. *et al.* Body Fat Distribution and Risk of Incident and Regressed Nonalcoholic Fatty Liver Disease. *Clinical gastroenterology and hepatology : the official clinical practice journal of the American Gastroenterological Association* **14**, 132-138.e134, doi:10.1016/j.cgh.2015.07.024 (2016). <https://doi.org/10.1016/j.cgh.2015.07.024>

29 Pickett-Blakely, O., Young, K. & Carr, R. M. Micronutrients in nonalcoholic fatty liver disease pathogenesis. *Cellular and Molecular Gastroenterology and Hepatology* **6**, 451-462 (2018). <https://doi.org/10.1016/j.jcmgh.2018.07.004>

30 Van Tien, N., Arisawa, K., Uemura, H. & Imaeda, N. Association Between Nutrient Patterns and Fatty Liver Index: Baseline Survey of the Japan Multi-Institutional Collaborative Cohort Study in Tokushima, Japan. **32**, 376-383, (2022). <https://doi.org/10.2188/jea.JE20200447>

31 Zaki, M., Amin, D. & Mohamed, R. Body composition, phenotype and central obesity indices in Egyptian women with non-alcoholic fatty liver disease. *Journal of Complementary and Integrative Medicine* **18**, 385-390 (2021). <https://doi.org/10.1515/jcim-2020-0073>

32 Zhang, F.-L. *et al.* Strong Association of waist circumference (WC), body mass index (BMI), waist-to-height ratio (WHtR), and waist-to-hip ratio (WHR) with diabetes: A population-based cross-sectional study in Jilin province, China. *Journal of diabetes research* **2021** (2021). <https://doi.org/10.1155/2021/8812431>

33 Pouwels, S. *et al.* Non-alcoholic fatty liver disease (NAFLD): a review of pathophysiology, clinical management and effects of weight loss. *BMC endocrine disorders* **22**, 1-9 (2022).

34 Mirmiran, P., Amirhamidi, Z., Ejtahed, H.-S., Bahadoran, Z. & Azizi, F. Relationship between diet and non-alcoholic fatty liver disease: a review article. *Iranian journal of public health* **46**, 1007 (2017). <https://doi.org/10.1186/s12902-022-00980-1>

35 Reddy, P., Lent-Schochet, D., Ramakrishnan, N., McLaughlin, M. & Jialal, I. Metabolic syndrome is an inflammatory disorder: A conspiracy between adipose tissue and phagocytes. *Clinica Chimica Acta* **496**, 35-44 (2019). <https://doi.org/10.1016/j.cca.2019.06.019>

36 Yoo, E. R. *et al.* Diet quality and its association with nonalcoholic fatty liver disease and all‐cause and cause‐specific mortality. *Liver International* **40**, 815-824 (2020). <https://doi.org/10.1111/liv.14374>

37 Succurro, E. *et al.* Usefulness of hemoglobin A1c as a criterion to define the metabolic syndrome in a cohort of italian nondiabetic white subjects. *The American journal of cardiology* **107**, 1650-1655 (2011). <https://doi.org/10.1016/j.amjcard.2011.01.055>

38 Chen, C. *et al.* HbA1c may contribute to the development of non-alcoholic fatty liver disease even at normal-range levels. *Bioscience reports* **40** (2020). <https://doi.org/10.1042/BSR20193996>

39 Karakousis, N. D., Chrysavgis, L., Chatzigeorgiou, A., Papatheodoridis, G. & Cholongitas, E. Frailty in metabolic syndrome, focusing on nonalcoholic fatty liver disease. *Annals of Gastroenterology* **35**, 234 (2022). <https://doi.org/10.20524/aog.2022.0705>
