# Supplementary material for: Path analysis model to identify the effect of poor diet quality on NAFLD among Iranian adults from Amol Cohort Study
Source: Sci Rep. 2024 Aug 27;14:19935. doi: 10.1038/s41598-024-70181-4 (PMC11358441; doi:10.1038/s41598-024-70181-4)
Supplement: Supplementary file 2 — Supplementary Table S1. [file 41598_2024_70181_MOESM2_ESM.docx]

Supplementary Info:

**Path analysis model to identify the effect of poor diet quality on NAFLD among Iranian adults from Amol Cohort Study**

Azam Doustmohammadian^1^, Bahareh Amirkalali ^1^, Barbora de Courten ^2^, Saeed Esfandyari ^3^, Nima Motamed^4^, Mansooreh Maadi^1^, Hossein Ajdarkosh^1^, Esmaeel Gholizadeh^1^, Samira Chaibakhsh^5^, Farhad Zamani^1^*

^1^ Gastrointestinal and Liver Diseases Research Center, Iran University of Medical Sciences, Tehran, Iran

^2^ School of Health and Biomedical Sciences, RMIT University, Melbourne, VIC 3085, Australia

^3^Asadabad School of Medical Sciences, Hamadan, Iran

^4^ Department of Social Medicine, Zanjan University of Medical Sciences, Zanjan, Iran

^5^Eye Research Center, The Five Senses Health Institute, Rassoul Akram Hospital, Iran University of Medical Sciences, Tehran, Iran.

*Correspondence to: Farhad Zamani

## Table S1. Adjusted means (SD)^a^ for Baseline characteristics across tertiles of HEI-2015 and NRF9.3 in the participants of Amol Cohort Study (n=2956)

| **Variables** | **HEI-2015** | | | | **NRF9.3** | | | |
| --- | --- | --- | --- | --- | --- | --- | --- | --- |
|  | **Tertile 1** | **Tertile 2** | **Tertile 3** | **p.value** | **Tertile 1** | **Tertile 2** | **Tertile 3** | **p.value** |
| **Women** |  |  |  |  |  |  |  |  |
| **Age (years)** | 46.34 ± 14.30 | 46.11± 13.55 | 45.50± 13.82 | 0.67 | 44.89 ± 14.51 | 46.78 ± 13.83 | 46.24 ± 13.36 | 0.12 |
| **BMI (kg/m2)** | 29.92± 5.51 | 29.49 ± 5.25 | 29.42 ± 4.95 | 0.30 | 29.59 ± 5.36 | 29.51± 5.11 | 29.76 ± 5.33 | 0.76 |
| **Waist circumference (cm)** | 89.58 ± 12.15 | 88.65± 12.35 | 86.18 ± 10.75 | < 0.001 | 87.49 ± 12.02 | 89.08 ± 12.18 | 88.19 ± 11.55 | 0.14 |
| **Smoker, n (%)** | 1(0.2) | 4(0.8) | 2(0.5) | 0.43 | 3(0.8) | 2(0.4) | 2(0.4) | 0.75 |
| **Alcohol drinker, n (%)** | 1(0.2) | 3(0.6) | 1(0.3) | 0.55 | 3(0.8) | 1(0.3) | 1(0.3) | 0.34 |
| **Diabetes, n (%)** | 108(23.0) | 101(20.7) | 54(14.4) | 0.006 | 72(18.0) | 87(19.0) | 104(21.9) | 0.30 |
| **Metabolic syndrome, n (%)** | 175(37.3) | 188(38.4) | 118(31.6) | 0.09 | 142(35.6) | 175(38.1) | 164(34.6) | 0.51 |
| **Heart disease, n (%)** | 25(5.3) | 16(3.3) | 9(2.4) | 0.06 | 19(4.8) | 14(3.1) | 17(3.6) | 0.40 |
| **Lowering serum glucose agent’s user, n (%)** | 24(5.4) | 44(9.4) | 15(4.0) | 0.004 | 16(4.1) | 31(7.0) | 36(8.0) | 0.06 |
| **Lowering serum lipid agent’s user, n (%)** | 66(14.3) | 87(18.0) | 36(9.6) | 0.002 | 37(9.3) | 59(12.9) | 93(20.0) | < 0.001 |
| **Lowering hypertension agent’s user, n (%)** | 92(19.6) | 121(24.7) | 71(19.0) | 0.06 | 81(20.3) | 83(18.1) | 120(25.3) | 0.02 |
| **Residual areas, n (%)** |  |  |  | < 0.001 |  |  |  | < 0.001 |
| Rural | 145(30.9) | 113(23.1) | 208(55.6) |  | 182(45.6) | 148(32.2) | 136(28.7) |  |
| Urban | 324(69.1) | 376(76.9) | 166(44.4) |  | 217(54.4) | 311(67.8) | 338(71.3) |  |
| **PA (MET-h/d), n (%)** |  |  |  | 0.03 |  |  |  | 0.54 |
| Very low | 133(28.6) | 123(25.3) | 85(22.8) |  | 98(24.6) | 125(27.3) | 118(25.2) |  |
| Low | 70(15.1) | 94(19.3) | 52(13.9) |  | 57(14.3) | 80(17.5) | 79(16.9) |  |
| Moderate | 156(33.5) | 168(34.6) | 159(42.6) |  | 152(38.2) | 167(36.5) | 164(35.0) |  |
| High | 106(22.8) | 101(20.8) | 77(20.6) |  | 91(22.9) | 86(18.8) | 107(22.9) |  |
| **Biochemical parameters** |  |  |  |  |  |  |  |  |
| TG (mg/dl) | 124.30 ± 70.36 | 135.92± 93.73 | 126.94± 104. | 0.11 | 132.81± 116.15 | 127.08± 68.17 | 128.51± 82.84 | 0.63 |
| Total Cholesterol(mg/dl) | 182.84± 42.27 | 183.37± 40.21 | 185.31± 42.58 | 0.67 | 186.24± 44.42 | 183.39± 38.06 | 181.94± 42.39 | 0.30 |
| HDL(mg/dl) | 46.40± 70.36 | 135.92± 93.73 | 126.94± 104.98 | 0.70 | 45.67± 13.82 | 46.22± 10.92 | 46.28± 10.86 | 0.71 |
| LDL(mg/dl) | 99.62± 26.03 | 99.80± 25.75 | 100.87± 28.11 | 0.76 | 99.62± 28.29 | 101.72± 24.24 | 98.75± 27.04 | 0.21 |
| SBP (mmHg) | 113.49± 20.05 | 113.50± 20.92 | 114.09± 20.48 | 0.89 | 114.80± 21.94 | 113.37± 20.08 | 112.98± 19.56 | 0.39 |
| DBP (mmHg) | 70.57± 12.35 | 69.35± 11.73 | 72.72± 12.58 | < 0.001 | 72.13± 12.94 | 69.69± 11.39 | 70.55± 12.40 | 0.01 |
| FBS (mg/dl) | 110.96± 41.07 | 109.65± 44.49 | 104.93± 34.06 | 0.08 | 108.31± 40.33 | 108.04± 41.08 | 109.91± 40.48 | 0.75 |
| HbA1C | 4.63± 0.91 | 4.67± 0.90 | 4.51± 1.01 | 0.04 | 4.63± 0.95 | 4.61± 0.91 | 4.59± 0.96 | 0.81 |
| CRP^c^ (mg/l) | 2.40 (1.00, 5.15) | 2.10 (1.00, 5.05) | 2.00(1.00, 5.00) | 0.41 | 2.40 (1.00, 5.00) | 2.00(1.00, 5.30) | 2.00 (1.00, 5.05) | 0.55 |
| ALT (mg/dl) | 20.06± 13.16 | 19.92± 12.78 | 19.82± 16.35 | 0.97 | 20.00± 14.48 | 19.66± 12.76 | 20.17± 14.71 | 0.85 |
| AST (mg/dl) | 20.1710± 10.32 | 19.3012± 6.66 | 19.3404± 7.70 | 0.20 | 19.6281± 8.13 | 19.27± 6.69 | 19.93± 9.96 | 0.48 |
| GGT (mg/dl) | 24.69± 19.68 | 24.04± 19.39 | 24.26± 17.54 | 0.86 | 23.71± 14.56 | 23.99± 19.44 | 25.19± 21.65 | 0.46 |
| ALKP (mg/dl) | 199.46± 71.68 | 195.22± 62.87 | 195.66± 69.69 | 0.58 | 197.35± 72.33 | 196.61± 59.13 | 196.62± 72.21 | 0.98 |
| **Dietary parameters** |  |  |  |  |  |  |  |  |
| DED | 1.60± 0.47 | 1.45± 0.29 | 1.30± 0.35 | < 0.001 | 1.52± 0.43 | 1.50± 0.37 | 1.37± 0.37 | < 0.001 |
| **Men** |  |  |  |  |  |  |  |  |
| **Age (years)** | 46.97 ± 15.66 | 47.61± 14.33 | 49.83± 14.93 | 0.004 | 47.82 ± 15.40 | 48.65 ± 14.94 | 47.97 ± 14.79 | 0.61 |
| **BMI (kg/m2)** | 26.98± 4.29 | 26.79 ± 4.17 | 26.70 ± 4.45 | 0.54 | 26.83 ± 4.64 | 26.75 ± 4.20 | 26.90 ± 4.31 | 0.83 |
| **Waist circumference (cm)** | 90.10± 10.20 | 89.98± 10.29 | 89.26 ± 10.61 | 0.35 | 89.23 ± 10.74 | 89.95 ± 10.09 | 90.07 ± 10.34 | 0.37 |
| **Smoker, n (%)** | 131(24.5) | 130(24.9) | 161(28.4) | 0.26 | 151(30.3) | 144(24.2) | 127(23.9) | 0.03 |
| **Alcohol drinker, n (%)** | 64(12.0) | 48(9.2) | 63(11.1) | 0.33 | 56(11.2) | 56(9.4) | 63(11.8) | 0.39 |
| **Diabetes, n (%)** | 66(12.3) | 55(10.5) | 58(10.2) | 0.48 | 46(9.2) | 60(10.1) | 73(13.7) | 0.04 |
| **Metabolic syndrome, n (%)** | 92(17.2) | 113(21.6) | 114(20.1) | 0.18 | 97(19.5) | 117(19.7) | 105(19.7) | 0.99 |
| **Heart disease, n (%)** | 31(5.8) | 20(3.8) | 34(6.0) | 0.21 | 24(4.8) | 31(5.2) | 30(5.6) | 0.84 |
| **Lowering serum glucose agent’s user, n (%)** | 31(6.1) | 23(4.6) | 32(5.8) | 0.57 | 25(5.2) | 32(5.6) | 29(5.7) | 0.93 |
| **Lowering serum lipid agent’s user, n (%)** | 41(7.7) | 60(11.6) | 61(10.8) | 0.08 | 54(10.9) | 57(9.7) | 51(9.7) | 0.75 |
| **Lowering hypertension agent’s user, n (%)** | 103(19.3) | 76(14.6) | 89(15.7) | 0.09 | 85(17.1) | 103(17.3) | 80(15.0) | 0.53 |
| **Residual areas, n (%)** |  |  |  | < 0.001 |  |  |  | < 0.001 |
| Rural | 275(51.4) | 194(37.2) | 332(58.6) |  | 297(59.6) | 275(46.3) | 229(43.0) |  |
| Urban | 260(48.6) | 328(62.8) | 235(41.4) |  | 201(40.4) | 319(53.7) | 303(57.0) |  |
| **PA (MET-h/d), n (%)** |  |  |  | 0.11 |  |  |  | 0.79 |
| Very low | 122(22.9) | 118(22.7) | 129(22.8) |  | 113(22.7) | 129(21.9) | 127(24.0) |  |
| Low | 69(12.9) | 70(13.5) | 59(10.4) |  | 56(11.2) | 71(12.0) | 71(13.4) |  |
| Moderate | 203(38.1) | 162(31.2) | 210(37.1) |  | 185(37.1) | 206(34.9) | 184(34.7) |  |
| High | 139(26.1) | 169(32.6) | 168(29.7) |  | 144(28.9) | 184(31.2) | 148(27.9) |  |
| **Biochemical parameters** |  |  |  |  |  |  |  |  |
| TG (mg/dl) | 137.05± 88.97 | 141.82± 86.18 | 137.30± 100.32 | 0.63 | 136.00± 81.31 | 140.89± 105.79 | 138.69± 85.40 | 0.68 |
| Total Cholesterol(mg/dl) | 177.83± 41.48 | 178.08± 36.93 | 178.35± 38.64 | 0.97 | 178.92± 37.53 | 177.64± 40.42 | 177.83± 38.93 | 0.84 |
| HDL(mg/dl) | 41.03± 11.39 | 41.67± 10.48 | 42.33± 12.18 | 0.16 | 41.77± 12.50 | 41.83± 11.39 | 41.45± 10.29 | 0.84 |
| LDL(mg/dl) | 97.52± 26.04 | 99.09± 25.30 | 99.43± 27.75 | 0.44 | 98.11± 26.35 | 99.63± 26.58 | 98.18± 26.31 | 0.55 |
| SBP (mmHg) | 116.12± 17.66 | 114.10± 17.20 | 117.69± 19.23 | 0.005 | 116.76± 20.00 | 115.69± 17.40 | 115.70± 17.08 | 0.55 |
| DBP (mmHg) | 72.38± 11.05 | 71.37± 10.56 | 73.29± 12.11 | 0.02 | 73.46± 11.64 | 72.26± 10.96 | 71.48± 11.29 | 0.01 |
| FBS (mg/dl) | 104.57± 31.29 | 102.19± 27.98 | 103.71± 29.22 | 0.41 | 101.80± 28.26 | 103.45± 31.01 | 105.17± 28.96 | 0.18 |
| HbA1C (%) | 4.55 ± 0.91 | 4.57± 0.88 | 4.50± 0.90 | 0.39 | 4.48± 0.93 | 4.60± 0.94 | 4.53± 0.80 | 0.08 |
| CRP^c^ (mg/l) | 1(0.50, 2.9) | 1.15(0.5, 3) | 1.1(0.5, 3.3) | 0.63 | 1.3(0.67, 3.5) | 1(0.50, 3.00) | 1 (0.50, 2.70) | 0.059 |
| ALT (mg/dl) | 28.69± 19.45 | 26.83± 17.67 | 26.78± 21.57 | 0.19 | 26.53± 17.32 | 28.25± 22.17 | 27.35± 18.81 | 0.35 |
| AST (mg/dl) | 23.46± 9.09 | 23.12± 9.53 | 23.49± 15.00 | 0.84 | 23.1239± 10.30 | 23.2013± 10.18 | 23.7836± 14.03 | 0.60 |
| GGT (mg/dl) | 30.32± 20.95 | 28.93± 18.68 | 29.08± 18.05 | 0.43 | 28.37± 17.39 | 29.93± 21.09 | 29.91± 18.74 | 0.32 |
| ALKP (mg/dl) | 201.16± 53.05 | 197.75± 51.21 | 198.81± 51.67 | 0.55 | 203.44± 54.30 | 196.94± 48.98 | 197.89± 52.83 | 0.09 |
| **Dietary parameter** |  |  |  |  |  |  |  |  |
| DED | 1.79± 0.64 | 1.53± 0.32 | 1.37± 0.35 | < 0.001 | 1.55± 0.45 | 1.65± 0.52 | 1.47± 0.48 | < 0.001 |

^a^Adjusted for energy obtained from analysis of covariance (ANCOVA), except for Dietary Energy Density (DED).

^b^ Significant difference between the first and last tertiles, obtained from Bonferroni’s post hoc test.

^c^CRP: C-reactive protein
