# Supplementary material for: Path analysis model to identify the effect of poor diet quality on NAFLD among Iranian adults from Amol Cohort Study
Source: Sci Rep. 2024 Aug 27;14:19935. doi: 10.1038/s41598-024-70181-4 (PMC11358441; doi:10.1038/s41598-024-70181-4)
Supplement: Supplementary file 3 — Supplementary Table S2. [file 41598_2024_70181_MOESM3_ESM.docx]

Supplementary Info:

**Path analysis model to identify the effect of poor diet quality on NAFLD among Iranian adults from Amol Cohort Study**

Azam Doustmohammadian^1^, Bahareh Amirkalali ^1^, Barbora de Courten ^2^, Saeed Esfandyari ^3^, Nima Motamed^4^, Mansooreh Maadi^1^, Hossein Ajdarkosh^1^, Esmaeel Gholizadeh^1^, Samira Chaibakhsh^5^, Farhad Zamani^1^*

^1^ Gastrointestinal and Liver Diseases Research Center, Iran University of Medical Sciences, Tehran, Iran

^2^ School of Health and Biomedical Sciences, RMIT University, Melbourne, VIC 3085, Australia

^3^Asadabad School of Medical Sciences, Hamadan, Iran

^4^ Department of Social Medicine, Zanjan University of Medical Sciences, Zanjan, Iran

^5^Eye Research Center, The Five Senses Health Institute, Rassoul Akram Hospital, Iran University of Medical Sciences, Tehran, Iran.

*Correspondence to: Farhad Zamani

## Table S2. Adjusted means (SD)^a^ for Dietary intakes across Tertiles (T) HEI2015 and NRF9.3 in the participants of Amol Cohort Study (n=2956)

|  | **HEI-2015** | | | | **NRF9.3** | | | |
| --- | --- | --- | --- | --- | --- | --- | --- | --- |
|  | **Tertile 1** | **Tertile 2** | **Tertile 3** | **p.value** | **Tertile 1** | **Tertile 2** | **Tertile 3** | **p.value** |
| **Women** |  |  |  |  |  |  |  |  |
| **Components of HEI_2015_** |  |  |  |  |  |  |  |  |
| **Adequacy components (score)** |  |  |  |  |  |  |  |  |
| Total fruits (5) | 3.98± 1.21 | 4.48± 0.89 | 4.78± 0.57 | < 0.001 | 4.14± 1.14 | 4.38± 0.96 | 4.61± 0.85 | < 0.001 |
| Whole fruits (5) | 4.76± 0.67 | 4.93± 0.33 | 4.98± 0.14 | < 0.001 | 4.83± 0.55 | 4.90± 0.44 | 4.92± 0.39 | 0.008 |
| Total vegetables (5) | 4.61± 0.84 | 4.84± 0.47 | 4.90± 0.31 | < 0.001 | 4.68± 0.72 | 4.77± 0.62 | 4.87± 0.48 | < 0.001 |
| Greens and beans (5) | 3.81± 1.54 | 4.30± 1.20 | 4.60± 0.89 | < 0.001 | 3.95± 1.43 | 4.11± 1.35 | 4.52± 1.04 | < 0.001 |
| Whole grains (10) | 6.22± 3.98 | 8.51± 2.72 | 8.74± 2.61 | < 0.001 | 8.03± 3.13 | 8.04± 3.34 | 7.29± 3.61 | 0.001 |
| Dairy (10) | 7.52±2.77 | 7.71±2.37 | 7.92±2.34 | < 0.001 | 7.73±2.58 | 7.64±2.50 | 7.74±2.48 | < 0.001 |
| Total protein foods (5) | 3.83± 1.31 | 4.06± 1.08 | 4.33±0.92 | < 0.001 | 4.02± 1.13 | 4.12± 1.11 | 4.01±1.18 | 0.28 |
| Seafood and plant proteins (5) | 1.82± 1.34 | 2.45± 1.46 | 2.87± 1.60 | < 0.001 | 2.33± 1.50 | 2.24± 1.52 | 2.45± 1.53 | 0.10 |
| Fatty acids (10) | 5.60± 1.67 | 5.93± 1.55 | 6.44± 1.75 | < 0.001 | 6.00± 1.84 | 5.93± 1.60 | 5.95± 1.62 | 0.81 |
| **Moderation components (score)** |  |  |  |  |  |  |  |  |
| Refined grains (10) | 9.11± 2.12 | 9.71± 1.02 | 9.45± 1.56 | < 0.001 | 9.39± 1.71 | 9.53± 1.51 | 9.31± 1.87 | 0.14 |
| Sodium (10) | 7.81± 2.83 | 8.60± 2.01 | 9.20± 1.78 | < 0.001 | 8.43± 2.51 | 8.18± 2.46 | 8.78± 2.02 | < 0.001 |
| Added sugars (10) | 9.71± 0.84 | 9.26± 1.42 | 6.87± 3.10 | < 0.001 | 7.86± 2.91 | 9.19± 1.74 | 9.43± 1.54 | < 0.001 |
| Saturated fats (10) | 4.41± 3.23 | 5.15± 2.96 | 6.63± 2.63 | < 0.001 | 3.90± 3.17 | 4.72± 3.21 | 5.41± 3.10 | < 0.001 |
| **Total score** | 66.67± 5.42 | 75.85± 1.87 | 86.85± 8.60 | < 0.001 | 77.80± 11.80 | 74.76± 8.86 | 74.84± 8.46 | < 0.001 |
| **Components of NRF9.3** |  |  |  |  |  |  |  |  |
| Protein (g/d) | 1.77± 0.39 | 1.68± 0.29 | 1.63± 0.30 | < 0.001 | 1.61± 0.34 | 1.74± 0.33 | 1.74± 0.33 | < 0.001 |
| Dietary fiber (g/d) | 1.32± 0.41 | 1.32± 0.30 | 1.33± 0.35 | 0.88 | 1.11± 0.28 | 1.30± 0.27 | 1.52± 0.38 | < 0.001 |
| Vitamin A (RAE) | 0.12± 0.09 | 0.12± 0.06 | 0.12± 0.07 | 0.50 | 0.11± 0.06 | 0.11± 0.06 | 0.14± 0.09 | < 0.001 |
| Vitamin C(mg/d) | 1.85± 0.92 | 1.88± 0.84 | 2.14± 1.03 | < 0.001 | 1.39± 0.52 | 1.72± 0.57 | 3.62± 1.07 | < 0.001 |
| Vitamin E (mg/d) | 0.49± 0.13 | 0.52± 0.15 | 0.52± 0.16 | < 0.001 | 0.51± 0.15 | 0.50± 0.15 | 0.52± 0.14 | 0.10 |
| Calcium (mg/d) | 1.16± 0.36 | 1.08± 0.28 | 1.06± 0.34 | < 0.001 | 0.98± 0.26 | 1.06± 0.28 | 1.24± 0.37 | < 0.001 |
| Iron (mg/d) | 1.18± 0.54 | 1.20± 0.44 | 1.27± 0.66 | 0.05 | 0.94± 0.22 | 1.11± 0.27 | 1.53± 0.74 | < 0.001 |
| Potassium (mg/d) | 1.06± 0.21 | 1.05± 0.18 | 1.08± 0.21 | 0.20 | 0.91± 0.13 | 1.02± 0.12 | 1.23± 0.20 | < 0.001 |
| Magnesium (mg/d) | 0.99± 0.19 | 1.02± 0.16 | 0.98± 0.15 | 0.004 | 0.90± 0.15 | 1.01± 0.15 | 1.07± 0.16 | < 0.001 |
| Added sugars (g/d) | 0.39± 0.31 | 0.55± 0.38 | 1.26± 0.93 | < 0.001 | 1.03± 0.34 | 0.61± 0.55 | 0.49± 0.48 | < 0.001 |
| Saturated fats (g/d) | 1.48± 0.39 | 1.36± 0.30 | 1.24± 0.32 | < 0.001 | 1.47± 0.38 | 1.36± 0.33 | 1.29± 0.32 | < 0.001 |
| Sodium (mg/d) | 1.03± 0.37 | 0.96± 0.42 | 0.90± 0.41 | < 0.001 | 1.01± 0.64 | 0.99± 0.26 | 0.91± 0.23 | 0.001 |
| **NR9** | 9.97± 1.79 | 9.91± 1.53 | 10.16± 2.14 | 0.12 | 8.48± 0.82 | 9.61± 0.65 | 11.65± 1.86 | < 0.001 |
| **LIM3** | 2.91± 0.57 | 2.88± 0.63 | 3.41± 1.11 | < 0.001 | 3.52± 1.04 | 2.97± 0.58 | 2.70± 0.55 | < 0.001 |
| **Total score** | 7.05± 1.93 | 7.02± 1.76 | 6.75± 2.37 | 0.05 | 4.96± 0.98 | 6.63± 0.40 | 8.94± 1.73 | < 0.001 |
| **Men** |  |  |  |  |  |  |  |  |
| **Components of HEI_2015_** |  |  |  |  |  |  |  |  |
| **Adequacy components (score)** |  |  |  |  |  |  |  |  |
| Total fruits (5) | 3.93± 1.22 | 4.49± 0.83 | 4.71± 0.62 | < 0.001 | 4.27± 1.02 | 4.28± 1.01 | 4.60± 0.86 | < 0.001 |
| Whole fruits (5) | 4.75± 0.68 | 4.94± 0.30 | 4.98± 0.14 | < 0.001 | 4.88± 0.47 | 4.88± 0.46 | 4.91± 0.40 | 0.44 |
| Dairy (10) | 6.84±2.82 | 7.45±2.36 | 7.56±2.31 | < 0.001 | 7.37±2.47 | 7.09±2.56 | 7.43±2.51 | < 0.001 |
| Total vegetables (5) | 4.47± 0.94 | 4.76± 0.56 | 4.85± 0.42 | < 0.001 | 4.66± 0.73 | 4.66± 0.71 | 4.77± 0.62 | 0.01 |
| Greens and beans (5) | 3.60± 1.58 | 4.25± 1.23 | 4.48± 1.03 | < 0.001 | 4.00± 1.39 | 4.00± 1.41 | 4.36± 1.19 | < 0.001 |
| Whole grains (10) | 5.60± 4.08 | 8.17± 3.02 | 8.65± 2.77 | < 0.001 | 7.67± 3.46 | 7.57± 3.60 | 7.23± 3.69 | 0.11 |
| Total protein foods (5) | 3.77± 1.28 | 4.11± 1.08 | 4.33±0.92 | < 0.001 | 4.11± 1.12 | 4.02± 1.14 | 4.10±1.12 | 0.31 |
| Seafood and plant proteins (5) | 1.67± 1.29 | 2.18± 1.42 | 3.01± 1.56 | < 0.001 | 2.25± 1.58 | 2.27± 1.50 | 2.38± 1.54 | 0.31 |
| Fatty acids (10) | 5.54± 1.61 | 5.84± 1.57 | 6.48± 1.62 | < 0.001 | 6.03± 1.81 | 6.00± 1.60 | 5.87± 1.52 | 0.24 |
| **Moderation components (score)** |  |  |  |  |  |  |  |  |
| Refined grains (10) | 9.02± 2.17 | 9.67± 1.17 | 9.57± 1.52 | < 0.001 | 9.49± 1.45 | 9.46± 1.60 | 9.31± 1.87 | 0.16 |
| Sodium (10) | 7.74± 2.78 | 8.71± 1.91 | 9.06± 1.99 | < 0.001 | 8.65± 2.45 | 8.22± 2.47 | 8.82± 1.96 | < 0.001 |
| Added sugars (10) | 9.76± 0.73 | 9.43± 1.22 | 7.05± 3.16 | < 0.001 | 7.20± 3.13 | 9.16± 1.64 | 9.21± 1.75 | < 0.001 |
| Saturated fats (10) | 3.77± 3.31 | 4.67± 2.96 | 5.97± 3.00 | < 0.001 | 4.80± 3.25 | 5.60± 3.03 | 2.80± 3.90 | < 0.001 |
| **Total score** | 65.73± 5.67 | 75.88± 1.93 | 87.31± 8.60 | < 0.001 | 80.36± 13.11 | 74.27± 9.23 | 75.46± 8.94 | < 0.001 |
| **Components of NRF9.3** |  |  |  |  |  |  |  |  |
| Protein (g/d) | 1.76± 0.58 | 1.73± 0.52 | 1.61± 0.29 | < 0.001 | 1.61± 0.54 | 1.72± 0.34 | 1.76± 0.55 | < 0.001 |
| Dietary fiber (g/d) | 1.29± 0.48 | 1.27± 0.30 | 1.32± 0.48 | 0.11 | 1.10± 0.30 | 1.29± 0.29 | 1.49± 0.56 | < 0.001 |
| Vitamin A(RAE) | 0.14± 0.13 | 0.12± 0.08 | 0.12± 0.08 | 0.04 | 0.11± 0.08 | 0.11± 0.07 | 0.15± 0.13 | < 0.001 |
| Vitamin C (mg/d) | 1.97± 1.11 | 1.95± 0.91 | 2.06± 1.07 | 0.11 | 1.42± 0.51 | 1.74± 0.54 | 2.81± 1.29 | < 0.001 |
| Vitamin E (mg/d) | 0.47± 0.23 | 0.50± 0.20 | 0.52± 0.31 | 0.006 | 0.48± 0.22 | 0.48± 0.13 | 0.51± 0.36 | 0.04 |
| Calcium (mg/d) | 1.14± 0.41 | 1.08± 0.33 | 1.00± 0.28 | < 0.001 | 0.95± 0.30 | 1.03± 0.27 | 1.23± 0.41 | < 0.001 |
| Iron (mg/d) | 1.19± 0.57 | 1.19± 0.45 | 1.24± 0.62 | 0.16 | 0.95± 0.32 | 1.12± 0.28 | 1.54± 0.76 | < 0.001 |
| Potassium (mg/d) | 1.05± 0.36 | 1.04± 0.18 | 1.04± 0.19 | 0.66 | 0.90± 0.13 | 1.00± 0.12 | 1.21± 0.35 | < 0.001 |
| Magnesium (mg/d) | 0.98± 0.28 | 1.01± 0.19 | 1.00± 0.33 | 0.15 | 0.91± 0.19 | 1.00± 0.15 | 1.08± 0.40 | < 0.001 |
| Added sugars (g/d) | 0.41± 0.32 | 0.61± 0.41 | 1.29± 0.91 | < 0.001 | 1.21± 0.96 | 0.61± 0.46 | 0.58± 0.49 | < 0.001 |
| Saturated fats (g/d) | 1.42± 0.50 | 1.34± 0.46 | 1.17± 0.27 | < 0.001 | 1.39± 0.50 | 1.28± 0.31 | 1.27± 0.46 | < 0.001 |
| Sodium (mg/d) | 1.06± 0.68 | 0.99± 0.90 | 0.91± 0.62 | 0.003 | 1.01± 1.03 | 0.98± 0.26 | 0.96± 0.77 | 0.52 |
| **NR9** | 10.01± 3.04 | 9.92± 1.96 | 9.95± 2.25 | 0.83 | 8.48± 1.50 | 9.54± 0.59 | 11.81± 3.21 | < 0.001 |
| **LIM3** | 2.90± 1.06 | 2.95± 1.34 | 3.37± 1.20 | < 0.001 | 3.61± 1.60 | 2.88± 0.51 | 2.81± 1.22 | < 0.001 |
| **Total score** | 7.10± 2.50 | 6.96± 1.81 | 6.57± 2.26 | < 0.001 | 4.86± 1.01 | 6.65± 0.40 | 9.00± 2.37 | < 0.001 |

^a^Adjusted for energy obtained from analysis of covariance (ANCOVA). ^b^ Significant difference between the first and last tertile obtained from Bonferroni’s post hoc test.
