# Supplementary material for: Path analysis model to identify the effect of poor diet quality on NAFLD among Iranian adults from Amol Cohort Study
Source: Sci Rep. 2024 Aug 27;14:19935. doi: 10.1038/s41598-024-70181-4 (PMC11358441; doi:10.1038/s41598-024-70181-4)
Supplement: Supplementary file 4 — Supplementary Table S3. [file 41598_2024_70181_MOESM4_ESM.docx]

Supplementary Info:

**Path analysis model to identify the effect of poor diet quality on NAFLD among Iranian adults from Amol Cohort Study**

Azam Doustmohammadian^1^, Bahareh Amirkalali ^1^, Barbora de Courten ^2^, Saeed Esfandyari ^3^, Nima Motamed^4^, Mansooreh Maadi^1^, Hossein Ajdarkosh^1^, Esmaeel Gholizadeh^1^, Samira Chaibakhsh^5^, Farhad Zamani^1^*

^1^ Gastrointestinal and Liver Diseases Research Center, Iran University of Medical Sciences, Tehran, Iran

^2^ School of Health and Biomedical Sciences, RMIT University, Melbourne, VIC 3085, Australia

^3^Asadabad School of Medical Sciences, Hamadan, Iran

^4^ Department of Social Medicine, Zanjan University of Medical Sciences, Zanjan, Iran

^5^ Echocardiography Research Center, Rajaie Cardiovascular Medical and Research Center, Iran University of Medical Sciences, Tehran, Iran

*Correspondence to: Farhad Zamani

**Table S3: Direct, indirect, and total coefficients path analysis for HEI-2015 and NRF9.3 in adult participants of AmolCS (n 2956), Iran, 2016–2017**

|  | | **Direct effect** | | | **Indirect effect** | | | **Total effect** | | |
| --- | --- | --- | --- | --- | --- | --- | --- | --- | --- | --- |
| **Endogenous variables** | **Exogenous variables** | **β** | **P-value^*^** | **95%CI** | **β** | **P-value^*^** | **95%CI** | **β** | **P-value^*^** | **95%CI** |
| **HEI_2015_** | | | | | | | | | | |
| Age | PA | -0.047 | **0.007** | -0.080, -0.013 | - | - | - | -0.047 | **0.007** | -0.00, -0.013 |
| Age | WHtR | 0.402 | **0.004** | 0.373, 0.438 | 0.001 | 0.703 | -0.003, 0.005 | 0.403 | **0.003** | 0.374, 0.439 |
| Age | HBA1c | 0.145 | **0.013** | 0.102, 0.186 | 0.043 | **0.008** | 0.026, 0.058 | 0.188 | **0.007** | 0.150, 0.229 |
| Age | CRP | 0.008 | 0.782 | -0.039, 0.051 | 0.072 | **0.007** | 0.052, 0.094 | 0.080 | **0.010** | 0.044, 0.118 |
| Age | MetS | 0.049 | **0.014** | 0.012, 0.077 | 0.199 | **0.004** | 0.181, 0.223 | 0.248 | **0.018** | 0.213, 0.279 |
| Age | NAFLD | -0.117 | **0.016** | -0.152, -0.082 | 0.207 | **0.009** | 0.185, 0.228 | 0.090 | **0.007** | 0.049, 0.130 |
| PA | WHtR | -0.049 | **0.008** | -0.082, -0.023 | -0.001 | 0.648 | -0.004, 0.002 | -0.049 | **0.013** | -0.082, -0.018 |
| HEI_2015_ | WHtR | -0.079 | **0.005** | -0.125, -0.052 | - | - | - | -0.079 | **0.005** | -0.125, -0.052 |
| HEI_2015_ | HBA1c | -0.033 | **0.037** | -0.072, -0.002 | -0.009 | **0.020** | -0.014, -0.005 | -0.042 | **0.004** | -0.082, -0.009 |
| HEI_2015_ | MetS | 0.025 | 0.072 | -0.004, 0.056 | -0.040 | **0.004** | -0.062, -0.026 | -0.015 | **0.004** | -0.049, 0.018 |
| WHtR | HBA1c | 0.108 | **0.011** | 0.065, 0.142 | - | - | - | 0.108 | **0.011** | 0.065, 0.142 |
| WHtR | CRP | 0.178 | **0.010** | 0.130, 0.231 | - | - | - | 0.178 | **0.010** | 0.130, 0.231 |
| WHtR | MetS | 0.409 | **0.009** | 0.379, 0.440 | 0.026 | **0.008** | 0.017, 0.037 | 0.435 | **0.010** | 0.404, 0.462 |
| WHtR | NAFLD | 0.394 | **0.012** | 0.355, 0.434 | 0.078 | **0.004** | 0.061, 0.100 | 0.472 | **0.006** | 0.442, 0.513 |
| MetS | NAFLD | 0.172 | **0.007** | 0.134, 0.213 | - | - | - | 0.172 | **0.007** | 0.134, 0.213 |
| **NRF_9.3_** | | | | | | | | | | |
| Age | PA | -0.047 | **0.007** | -0.080, -0.013 | **-** | - | - | -0.047 | **0.007** | -0.080, -0.013 |
| Age | WHtR | 0.400 | **0.013** | 0.372, 0.436 | 0.002 | **0.010** | 0.001, 0.005 | 0.402 | **0.003** | 0.374, 0.439 |
| Age | HBA1c | 0.143 | **0.016** | 0.095, 0.182 | 0.045 | **0.007** | 0.030, 0.060 | 0.188 | **0.007** | 0.150, 0.229 |
| Age | CRP | 0.007 | 0.778 | -0.039, 0.051 | 0.073 | **0.006** | 0.053, 0.096 | 0.080 | **0.010** | 0.044, 0.118 |
| Age | MetS | 0.050 | **0.015** | 0.013, 0.077 | 0.198 | **0.004** | 0.180, 0.222 | 0.248 | **0.018** | 0.213, 0.279 |
| Age | NAFLD | -0.119 | **0.016** | -0.154, -0.081 | 0.209 | **0.006** | 0.188, 0.232 | 0.090 | **0.007** | 0.049, 0.130 |
| PA | WHtR | -0.049 | **0.013** | -0.082, -0.019 | - | - | - | -0.049 | **0.013** | -0.082, -0.018 |
| NRF_9.3_ | NAFLD | -0.090 | **0.010** | -0.119, -0.059 | -0.005 | 0.514 | -0.023, 0.010 | -0.095 | **0.015** | -0.125, -0.057 |
| WHtR | HBA1c | 0.111 | **0.009** | 0.069, 0.146 | - | - | - | 0.111 | **0.009** | 0.069, 0.146 |
| WHtR | CRP | 0.178 | **0.012** | 0.126, 0.228 | - | - | - | 0.178 | **0.012** | 0.126, 0.228 |
| WHtR | MetS | 0.407 | **0.010** | 0.377, 0.437 | 0.026 | **0.007** | 0.017, 0.037 | 0.433 | **0.010** | 0.403, 0.459 |
| WHtR | NAFLD | 0.396 | **0.008** | 0.359, 0.438 | 0.077 | **0.004** | 0.059, 0.099 | 0.473 | **0.005** | 0.443, 0.512 |
| HBA1c | MetS | 0.156 | **0.007** | 0.116, 0.194 | - | - | - | 0.156 | **0.007** | 0.116, 0.194 |
| HBA1c | NAFLD | 0.037 | **0.023** | 0.007, 0.064 | 0.027 | **0.007** | 0.018, 0.037 | 0.064 | **0.019** | 0.029, 0.092 |
| CRP | MetS | 0.048 | **0.005** | 0.017, 0.095 | - | - | - | 0.048 | **0.005** | 0.017, 0.095 |
| MetS | NAFLD | 0.170 | **0.005** | 0.136, 0.212 | - | - | - | 0.170 | **0.005** | 0.136, 0.212 |

Abbreviations: PA; physical activity; NAFLD: nonalcoholic fatty liver disease; HEI_2015_: healthy eating index 2015; NRF_9.3_; nutrient-rich food 9.3; WHtR: waist-to-height ratio; HBA1c: hemoglobin A_1c_; CRP: c-reactive protein; CI: confidence intervals; MetS: metabolic syndrome

Note: Statistically non-significant total effects are not reported.
